# Feature co-localization landscape of the human genome

**DOI:** 10.1038/srep20650

**Published:** 2016-02-08

**Authors:** Siu-Kin Ng, Taobo Hu, Xi Long, Cheuk-Hin Chan, Shui-Ying Tsang, Hong Xue

**Affiliations:** 1 Division of Life Science, Applied Genomics Center and Center for Statistical Science, Hong Kong University of Science and Technology, Clear Water Bay, Hong Kong

## Abstract

Although feature co-localizations could serve as useful guide-posts to genome architecture, a comprehensive and quantitative feature co-localization map of the human genome has been lacking. Herein we show that, in contrast to the conventional bipartite division of genomic sequences into genic and inter-genic regions, pairwise co-localizations of forty-two genomic features in the twenty-two autosomes based on 50-kb to 2,000-kb sequence windows indicate a tripartite zonal architecture comprising Genic zones enriched with gene-related features and Alu-elements; Proximal zones enriched with MIR- and L2-elements, transcription-factor-binding-sites (TFBSs), and conserved-indels (CIDs); and Distal zones enriched with L1-elements. Co-localizations between single-nucleotide-polymorphisms (SNPs) and copy-number-variations (CNVs) reveal a fraction of sequence windows displaying steeply enhanced levels of SNPs, CNVs and recombination rates that point to active adaptive evolution in such pathways as immune response, sensory perceptions, and cognition. The strongest positive co-localization observed between TFBSs and CIDs suggests a regulatory role of CIDs in cooperation with TFBSs. The positive co-localizations of cancer somatic CNVs (CNVT) with all Proximal zone and most Genic zone features, in contrast to the distinctly more restricted co-localizations exhibited by germline CNVs (CNVG), reveal disparate distributions of CNVTs and CNVGs indicative of dissimilarity in their underlying mechanisms.

The focus of molecular genetic studies of complex human phenotypes have shifted during recent years, from a candidate-gene approach based on a priori knowledge of gene functions to an open approach of genome-wide association studies (GWAS)^1^,^2^. SNPs are widely employed as markers in Common-Disease-Common-Variant (CDCV) strategy-based association studies^3^,^4^,^5^. However, because SNPs explain only a small fraction of the phenotypic variations in complex traits or diseases, a Common-Disease-Rare-Variant (CDRV) strategy focusing on rare sequence and structural variants has been proposed^6^,^7^,^8^. While the application of rare variants could be limited by their rarity, the difficulty may be overcome by massive parallel sequencing^9^,^10^,^11^.

Since the utility of markers depends on not only their frequencies but also their distances from target sequences, it becomes important to assess the frequencies of different markers at locations relative to genic and regulatory sequences. As well, the human genome contains a multitude of functionally unannotated but highly conserved and therefore presumably functional sequences, and the co-localizations of such sequences with known functional features could facilitate their annotation. In this regard, the human genome comprises a wide range of functional and structural features, and painstaking research has enabled the construction of extensive databases on the locations of various features. Moreover, different kinds of features may not be located independently of one another, as exemplified by the positive co-localization of genes and Alu transposons^12^,^13^, and the negative co-localization between genes and AT-rich regions^14^,^15^. Accordingly, the present study has been directed to examine the pairwise co-localizations of forty-two features of the genome documented in public databases. The propensities for individual pairs of features to co-localize either positively or negatively, measured based on the sign and strength of the correlations between their occurrences in non-overlapping sequence windows in twenty-two autosomes, reveal a zonal architecture of the human genome characterized by three types of zones, and a number of unexpected pairwise feature co-localizations with functional implications. Moreover, numerous CNV and SNP hotspots are found to be co-localized in the same sequence windows, suggesting the application of a new Common-Disease-Joint-Variants (CDJV) strategy for complex trait or disease association studies.

## Results

### Co-localization based grouping of genomic features

**Figure 1:**
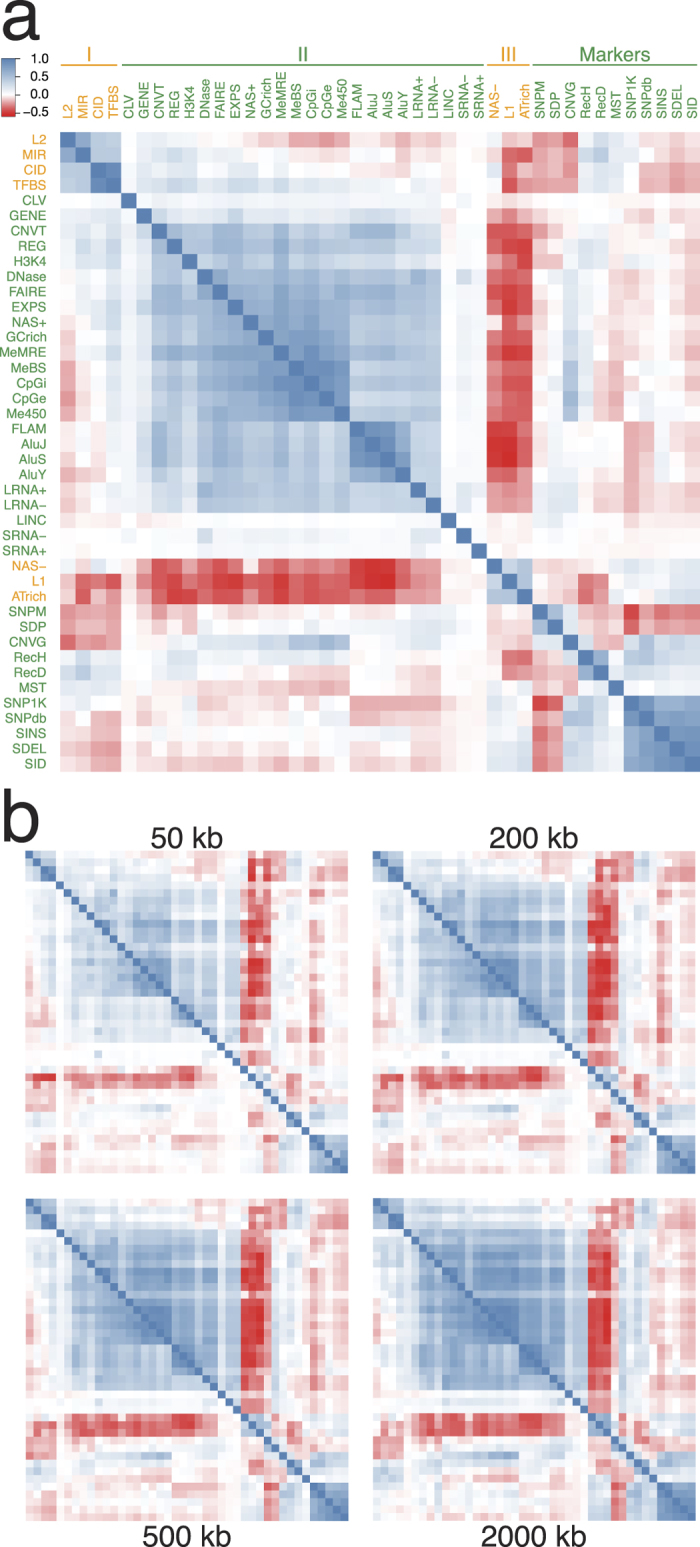
Co-localizations among genomic features. (**a**) Heat map of pairwise correlation coefficient *r* among forty-two genomic features in 500-kb non-overlapping windows in the twenty-two autosomes. L2 *long interspersed nuclear element 2*, MIR *mammalian-wide interspersed repeat*, CID *conserved indel*, TFBS *conserved transcription factor binding site*, CLV *breakpoints of variants in ClinVar database*, GENE *known gene sequences*, CNVT *somatic CNV breakpoints*, REG *published DNA regulatory regions*, H3K4 *histone 3 lysine 4 trimethylation*, DNase *open chromatin elements and signals*, FAIRE *formaldehyde-assisted isolation of regulatory element*, EXPS *gene expression data*, NAS+ *nuclease accessible sites in CD34*+ *cells*, GCrich *GC-rich DNA sequences*, MeMRE *genome wide methylation data*, MeBS *CpG methylation*, CpGi *CpG island*, CpGe *evolutionarily CpG rich regions*, Me450 *CpG methylation*, FLAM *free left Alu monomer*, AluJ, AluS and AluY *sub-families of short interspersed nuclear element (SINE)*, LRNA+ *long RNA-seq signals of plus strand*, LRNA- *long RNA-seq signals of minus strand*, LINC *lincRNA*, SRNA- *short RNA-seq signals of minus strand*, SRNA+ *short RNA-seq signals of plus strand*, NAS- *nuclease accessible sites in CD34- cells*, L1 *long interspersed nuclear element 1*, ATrich *AT-rich DNA sequences*, SNPM *SNPs mapped to multiple locations*, SDP *segmental duplication*, CNVG *germline CNV breakpoints*, RecH *recombination rate from HapMap*, RecD *sex-averaged recombination rate from deCODE*, MST *microsatellite*, SNP1K *single nucleotide polymorphism in 1000 Genomes Project*, SNPdb *single nucleotide polymorphism in dbSNP*, SINS *small insertion in dbSNP*, SDEL *small deletion in dbSNP*, SID *small indel*. Positive and negative co-localizations are expressed in *r-*values based on the blue and red thermal scales respectively. The partitions of various features into Groups I-III and Markers were based on their nature and co-localization patterns, guided foremost by the strong positive co-localizations. (**b**) Heat map of pairwise Pearson correlation coefficients (upper triangle) and Spearman correlation coefficients (lower triangle) among genomic features in 50-kb, 200-kb, 500-kb and 2,000-kb windows. Arrangement of different features on x and y axes same as in (**a**).

**Figure 2:**
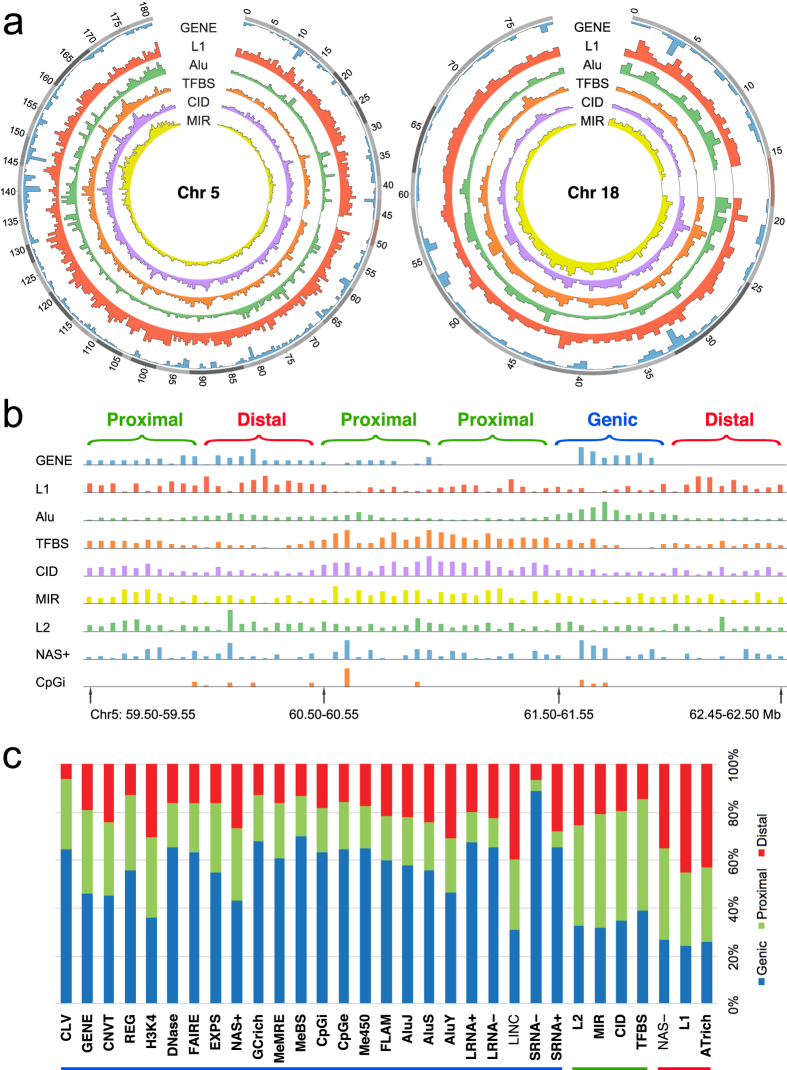
Distributions of genomic features among different sequence zones. (**a**) Circos diagrams for chromosomes 5 and 18 showing from outside inward positions on chromosome in Mb, and abundances (Supplementary Table S4) of GENE (blue), L1 (red), Alu viz. sum of AluY, AluS, AluJ and FLAM (green), TFBS (orange), CID (purple) and MIR (yellow) in 500-kb windows expressed in separate linear scales. (**b**) Distributions of a number of features in 50-kb windows in the 59.50–62.50 Mb segment of chromosome 5, and their partition into 500-kb Genic, Proximal and Distal windows. (**c**) Bar chart plotting fractional distributions of non-Marker features among Genic, Proximal and Distal zones (as given in Supplementary Table S5) against their zonal classification based on Supplementary Table S1. Twenty-nine of the 31 features yield a correct match: 23/24 Genic features (blue underlined) show largest fractional distribution in Genic zones (blue section of bar), 4/4 Proximal features (green underlined) show largest fractional distribution in Proximal zones (green section), and 2/3 Distal (red underlined) features show largest fractional distribution in Distal zones (red section). The names of these features with correct matching are marked by bold font. The classifications of LINC and NAS-, which do not yield a correct match, are also shown by Fig. 1a and Supplementary Table S2 to be borderline: LINC forms red squares with all Group I, all Group III and some but not all Group II features; and NAS- forms blue squares with three-out-of-four features in Group I, both L1 and ATrich in Group III, but red squares with all except one (viz. SRNA+) features in Group II. Notably, the high fractional distribution of 0.887 in Genic zones displayed by SRNA- indicates that the ‘Genic’ windows identified by the feature-ratios method are highly distinct from the identified ‘Proximal’ and ‘Distal’ windows. Likewise, the low fractional distribution of 0.065 in Proximal zones displayed by SRNA+ indicates that the ‘Proximal’ windows identified are highly distinct from the identified ‘Genic’ and ‘Distal’ windows.

In Fig. 1a, the strength of any pairwise co-localization among the forty-two genomic features (see Supplementary Table S1 for feature descriptions) is indicated by the correlation coefficient *r* on a thermal scale, in blue for positive and red for negative (viz. mutual exclusion) co-localizations. Group I features include long interspersed nuclear element-2 (LINE-2, or L2), mammalian wide interspersed repeats (MIRs), evolutionarily conserved and therefore presumably functional non-coding DNA in the form of CIDs^16^, and TFBSs form blue squares indicating positive co-localizations with one another. The inclusion of TFBS, together with the stronger co-localizations formed by Group I features with the regulatory regions in ORegAnno database (REG), regulatory elements isolated with formaldehyde assistance (FAIRE) and histone-3 lysine-4 trimethylation sites (H3K4) compared to genic sequences in RefSeqGene (GENE) indicate that Group I features are located near genic regions. That MIR and L2 form blue squares with TFBS as well as GENE, REG and H3K4 is in agreement with the proposal that MIR and L2 elements have been recruited to serve cellular functions^17^,^18^,^19^. Notably, the strong co-localization of TFBS and CID is evident not only with 500-kb windows (Fig. 1a), but also with 2,000, 200 or 50-kb windows analysed based on either Pearson or Spearman correlation coefficients (Fig. 1b; also in more details in Supplementary Fig. S1 and Supplementary Table S2). As well, the Circos diagrams of chromosomes 5 and 18 (Fig. 2a) and those of other autosomes (Supplementary Fig. S2) show the extensive overlaps between the TFBS and CID peaks.

Group II features are defined by a large block of blue squares pointing to the mutual positive co-localizations among features belonging to this group. The inclusion of GENE in this group indicates that the sequence windows enriched with Group II features represent the genic regions in the genome. That GENE in Group II forms a blue square with TFBS in Group I is in accord with the expected proximity between the locations of GENE and TFBS. Other features in Group II, including gene-expression data based on RNA-seq (EXPS), nuclease-accessible sites in CD34 +cells (NAS+), open chromatin elements and signals based on DNase sensitivity (DNase), GC rich regions (GCrich), and the Alu family of short interspersed nuclear elements (AluJ, AluS and AluY) together with free left Alu monomer (FLAM) are all known to be associated with genic regions. The same applies to the group of CpG- and methylation-related features: genome wide methylation based on methylation-sensitive restriction enzyme (MeMRE), CpG rich islands (CpGi), CpG rich regions inferred from evolutionary dynamics (CpGe), CpG methylation based on reduced representation bisulfite sequencing (MeBS) and CpG methylation based on 450 bead array (Me450). CNVT and variants in ClinVar database (CLV) are similar to GENE in their formation of positive co-localizations with numerous Group II features and TFBS but not with SNPs in dbSNP (SNPdb), SNPs in 1K-Genomes Project (SNP1K) or small indels in 1K-Genomes Project (SID) (Fig. 1a).

Group III features consisting of nuclease-accessible sites in CD34-negative cells (NAS-), LINE-1 elements (L1) and AT-rich regions (ATrich) form blue squares of co-localizations with one another but red squares with the large majority of Group I and Group II features, which places them in intergenic regions in accord with the suggestion that L1 elements are particularly abundant in AT-rich, gene-poor and low-recombining genomic regions^20^,^21^. The negative co-localization between L1 and GENE is also indicated by the circos diagrams of various autosomes (Fig. 2a; Supplementary Fig. S2), where L1 peaks are frequently most prominent at chromosomal locales relatively low in GENE occurrences. Since the lengths of L1 elements range up to only 8.5 kb^22^, the negative co-localizations exhibited by L1 toward TFBS, GENE, REG or EXPS within 500-kb or even 2,000-kb windows using either Pearson or Spearman method (Supplementary Fig. S1) appear to be ascribable more to mutual incompatibility than lack of sequence space within the windows.

### Co-localization patterns of marker features

**Figure 3:**
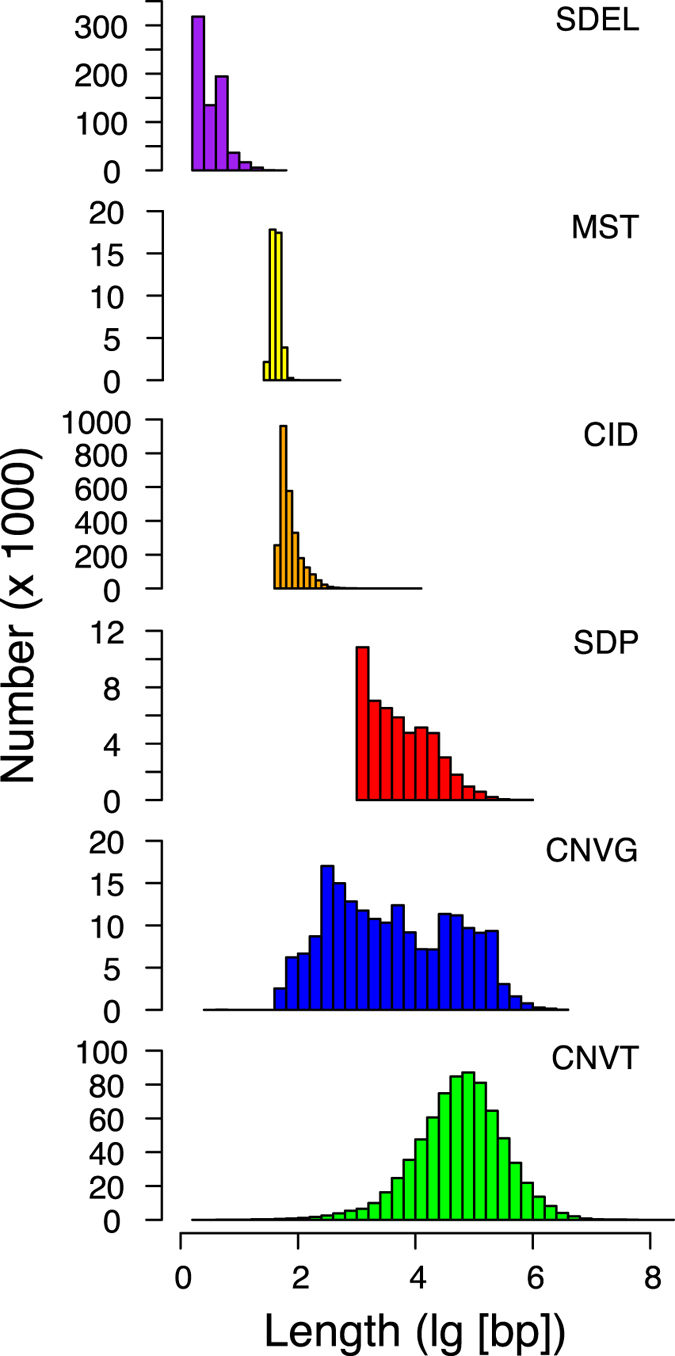
Length distribution of different types of DSVs. Length distributions of different types of DSVs. The different distribution profiles are arranged in ascending order of average length based on data given in Supplementary Table S3.

Marker features in Fig. 1 comprise those features that can be employed as tools in genetic studies, including recombination sites and DNA sequence variations (DSVs) with the exceptions of CID, CNVT and CLV which are allocated to Group I or II on account of their propensities for positive co-localizations with features in those groups. The divergence between the red squares formed by CNVG and the blue squares formed by CNVT toward Group I features is consistent with the dissimilarity between their fragment lengths (Fig. 3): CNVTs (medium length 57,260 bp, Supplementary Table S3) are much longer than CNVGs (medium length 2,784 bp)^23^. As well, the length distribution of somatic cancer CNVTs conforms closely to a normal curve, suggesting that they have not been subject to extensive selection. In contrast, the marked deviation of the length distribution of germline CNVG from a normal curve is suggestive of a history of extensive selection (Fig. 3). The strong blue squares formed by CNVG with MeMRE, MeBS, CpGi, CpGe and Me450 underlines the possible importance of CNVG toward regulation of methylation-related sites.

Interestingly, the small mutations, viz. SNPdb, SNP1K, small insertions from dbSNP (SINS), small deletions from dbSNP (SDEL) and SID form blue squares with one another and with Group III features, but with relatively few features in Groups I and II. The recombination sites from deCODE (RecD) based on MST^24^ form red squares with NAS+, MeBS, CpGi, CpGe and Me450 in keeping with the red squares formed by MST with these features. In contrast, recombination sites from HapMap (RecH) based on SNPs form blue squares with these same features. It is also notable that MST and CNVG tend to form co-localizations of opposite signs: MST forms largely blue squares with Groups I and III features and red squares with Group II features, whereas CNVGs forms largely red squares with Groups I and III features and blue squares with Group II features.

### Methodological effects on estimation of co-localizations

**Figure 4:**
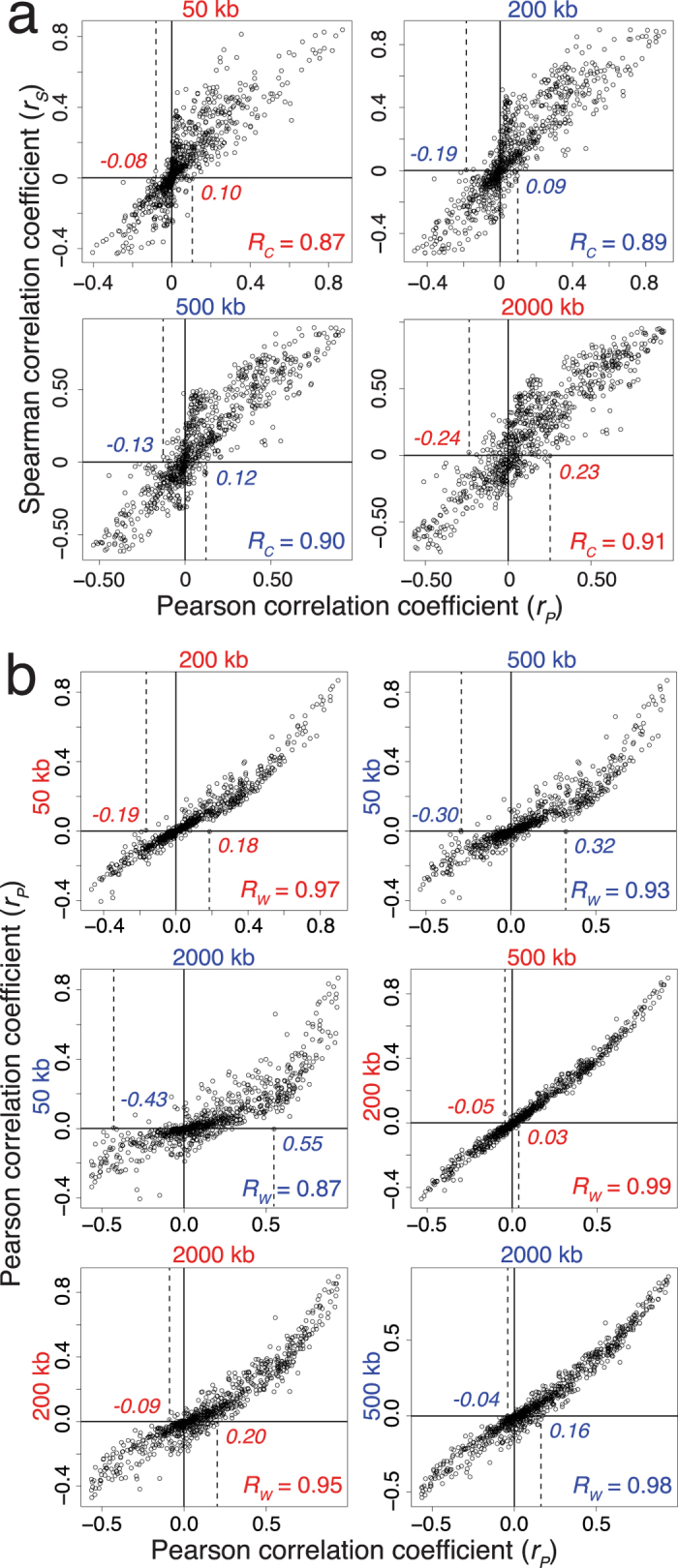
Effects of statistical methodology and window size on co-localization coefficients. (**a**) Scatterplots of Pearson and Spearman correlation coefficients for 861 pairwise co-localizations among 42 genomic features estimated using different window sizes. *R*_*C*_ the linear correlation between the two types of correlation coefficients is shown at lower right of panel. (**b**) Scatterplots of Pearson correlation coefficients for co-localizations estimated using pairs of window sizes. *R*_*W*_ the linear correlation coefficient between Pearson coefficients obtained with two window sizes is shown at lower right of panel. For the 500-kb window plot in (**a**), the vertical dashed lines indicate that all Pearson coefficients of >0.12 value correspond to positive Spearman coefficients, whereas all Pearson coefficients of <−0.13 value correspond to negative Spearman coefficients. Similar dashed lines in other plots in (**a**,**b**) indicate comparable correspondences either between Pearson and Spearman coefficients, or between Pearson coefficients obtained using two different window sizes. *R*_*C*_ and *R*_*W*_ were estimated using the ‘cor’ function with default option in R program.

In Fig. 1a, the pairwise co-localizations were estimated using 500-kb windows based on Pearson correlation coefficient *r*. To examine the effects of statistical methodology, pairwise co-localizations were estimated based on both Pearson and Spearman correlation coefficients for different window sizes (Supplementary Table S2). The results, shown in Fig. 4a, indicate that the linear correlation coefficient *R*_*C*_ between Spearman coefficients and Pearson coefficients ranged from 0.87 to 0.91 for the 50-kb, 200-kb, 500-kb and 2,000-kb windows. In Fig. 4b, the linear correlation coefficient *R*_W_ between Pearson coefficients for different window sizes ranged from 0.87 to 0.99, with the latter figure pertaining to the correlation between 200-kb and 500-kb windows. Therefore, there was a high degree of consistency between Spearman and Pearson coefficients, and between Pearson coefficients for different window sizes, pointing to the robustness of the *co-localization coefficients* (viz. pairwise correlation coefficient estimated by Pearson or Spearman method) with respect to methodology and the maximum stability of the coefficients obtained using 200-kb and 500-kb windows.

Accordingly, although use of different window sizes or choice of Pearson versus Spearman coefficients results in some variations in the co-localization coefficients, the positive or negative sign of Pearson correlation coefficient *r* remains invariant with window size over the 50–2,000 kb range for most co-localizations even though the magnitude of *r* in general decreases in the smaller windows; the average difference in *r* values between the 50-kb windows and forty-fold larger 2000-kb windows is only 13.8%. This stability in the sign of *r* stems from the highly significant correlations embodied in many of the pairwise co-localizations made possible by the large populations of the features analysed (with > 10,000 entries in every database in Supplementary Table S1).

### Prominent pairwise feature co-localizations

**Figure 5:**
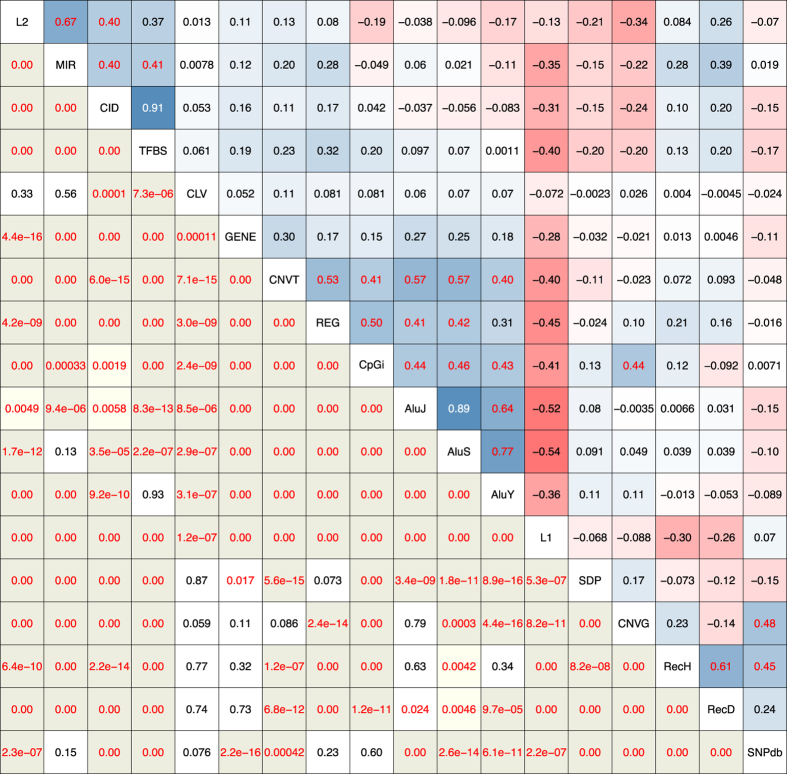
Parameters of prominent co-localizations. Pearson correlation coefficient *r*-values based on 500-kb windows in twenty-two autosomes are shown in the upper triangle in the thermal scales employed in Fig. 1a, with blue for positive and red for negative correlations, and *r*-values greater than 0.4 are shown in red font (except for the two highest *r*-values in white font). Asymptotic *P*-values are shown in the lower-left triangle. The feature named inside a box on the diagonal at the junction between a column and a row participates in all the pairwise co-localizations represented on the intersecting column and row. *P* ≥ 0.05 are shown in black font in white boxes, 0.05 > *P* ≥ 0.01 in red font in white boxes, 0.01 > *P* ≥ 0.001 in red font in light yellow boxes, *P* < 0.001 in red font in grey boxes; and *P* < 10^−16^ are shown as ‘0.00’. Estimation of *r* and *P* values is described under Methods, and similar co-localization parameters obtained using 50, 200 and 2,000-kb windows are shown in Supplementary Fig. S3.

The upper triangle in Fig. 5 shows that, for 500-kb windows, the CID-TFBS pair yields the highest *r*-value of 0.91, followed by the *r*-values for the L2-MIR pair and the pairings among the three Alu elements. The high *r* values exhibited by CNVT toward REG, CpGi and GENE attest to the concentration of somatic tumor CNVs at such functional sites. The statistical significance of many pairwise co-localizations is made evident by the asymptotic *P*-values in the lower triangle of the figure. There are only 20 out of 153 boxes with *P* > 0.05 compared to 84 boxes with *P* < 10^−16^ (shown as ‘0.00’). The positive ‘0.00’ co-localizations formed by the Alu elements toward REG and GENE indicate the highly significant enrichment of these elements in the vicinity of REG and GENE, as illustrated by the importance of an AluY-containing segment in *GABRB2* in schizophrenia etiology^25^, and the efficient capture of genic regions for sequence analysis by the AluScan platform developed by our laboratory based on inter-Alu polymerase chain reaction employing multiple Alu consensus sequence-based primers with opposite orientations^12^,^26^,^27^. Similar prominent co-localizations obtained with 50, 200 and 2,000-kb windows are shown in Supplementary Fig. S3.

### Zonal architecture based on feature co-localizations

**Figure 6:**
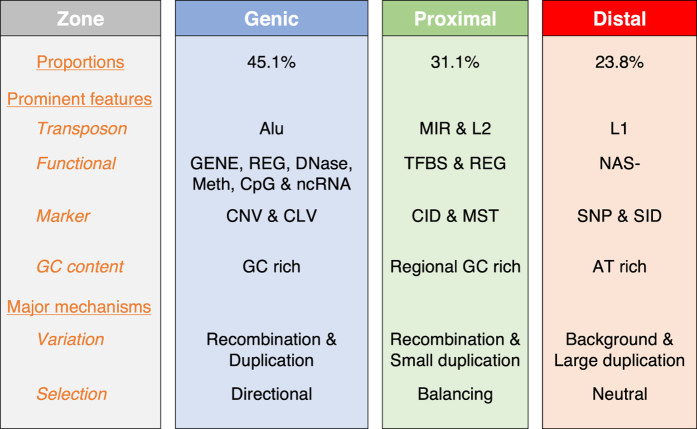
Major architectural zones in human genome. Sequence zones based on feature co-localization patterns include the Genic, Proximal and Distal zones, the constituent features of which are shown in Fig. 1. The characteristics of each zone are indicated in terms of its foremost functional features, transposable elements, markers, GC content, and major mechanisms for generation of sequence diversity and evolution. Preliminary analysis of the partition of non-gap 500-kb windows in the genome among the different zones yields 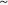
45.1% Genic, 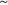
31.1% Proximal and 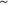
23.8% Distal zones (Supplementary Fig. S4). See Fig. 1 legend for abbreviations of features except for Alu = AluJ, AluS, AluY and FLAM; Meth = MeMRE, Me450 and MeBS; CpG = CpGi, CpGe; and ncRNA = LRNA+, LRNA-, SRNA+, SRNA- and LINC.

Genomic sequences are conventionally divided into genic and intergenic regions. However, the feature co-localizations determined in this study identified three major types of architectural zones in the human genome, viz. the Genic, Proximal and Distal zones (Fig. 6). Genic zones are enriched in Group II features which form the largest block of blue squares in Fig. 1a, and also in CNVGs that co-localize with promoter-related and epigenetic features, suggesting that CNVGs furnish population diversity for Genic zone sequences where directional selection is prevalent.

Proximal (i.e. gene-proximal) zones are enriched in the four mutually co-localizing Group I features L2, MIR, CID and TFBS. These features form red squares with CNVG, small indels (SINS, SDEL and SID) and SNPdb, but blue squares with RecH, RecD, MST and SNP1K. Previous evidence has indicated that the functional non-coding sequences in CIDs have been subjected to both positive selection with respect to substitutions and purifying selection with respect to indels^16^. This is consistent with the presence of balancing selection preserving both the insertion and deletion forms of the CIDs.

Distal (i.e. gene-distal) zones are enriched in the three Group III features NAS-, L1 and ATrich which form red squares with most other functional features, but form blue squares with one another and some marker features; while forming prominent red squares with RecH and RecD, they form blue squares with MST and the small sequence variations SNP1K, SNPdb, SINS, SDEL and SID. This suggests that Distal zones can tolerate small background variations, and are more subject to neutral evolution than Genic and Proximal zones.

The intermingled distribution and statistical nature of sequence windows belonging to the three types of zones are illustrated by the six 500-kb windows in the 59.50–62.50 Mb segment of chromosome 5 in Fig. 2b, where the densities or intensities of various features are shown for each 50-kb subdivision. The strong parallelism between the distributions of TFBS and CID, and their more modest parallelism with those of MIR and L2, are readily recognizable in the three Proximal zone windows, which nonetheless contain GENE, L1, Alu, NAS+ and CpGi. Similarly, the one Genic zone is enriched with Gene, Alu, NAS+ and CpGi, and the two Distal zones are both enriched with L1, but non-exclusively in all three cases.

The feature compositions of all the 500-kb windows on the 22 autosomes together with their classification into Genic, Proximal and Distal zone windows based on the feature-ratios method described in Supplementary Fig. 4 are given in Supplementary Table S4. On this basis, all but two (viz, LINC and NAS-) of the thirty-one Genic, Proximal and Distal zone features are expectedly most abundant in the Genic, Proximal and Distal windows respectively, thereby yielding a correct match between the zonal designation of each of these features and its most abundant zonal location (Fig. 2c, Supplementary Table S5). This 29/31, or 93.5% accuracy corroborates the utility of the feature-ratios method for classifying windows.

The proportions of Genic, Proximal and Distal windows of 45.1%, 31.1% and 23.8% respectively estimated by feature-ratios are in close agreement with the 44.4%, 31.5% and 24.1% proportions of SNPdb entries (containing mostly germline SNPs) found in these three types of zones, which supports a largely equal density of germline SNPs in the Genic, Proximal and Distal zones. In contrast, the proportion of SNPs in Genic zones is increased to 70.6% in the common clinical SNP subset of SNPdb, and to 65.6% in CLV, in accord with the expectation that Genic zones are more commonly involved in disease etiology than Distal zones if not Proximal zones. The proportion in Genic zones is also higher at 52.1% for the SNPs in NHGRI GWAS for complex phenotypes compared to 42.2% for the SNPs in Affymetrix 6.0 (Supplementary Fig. S5a–f).

### Co-localization of SNP-CNV hotspots

**Figure 7:**
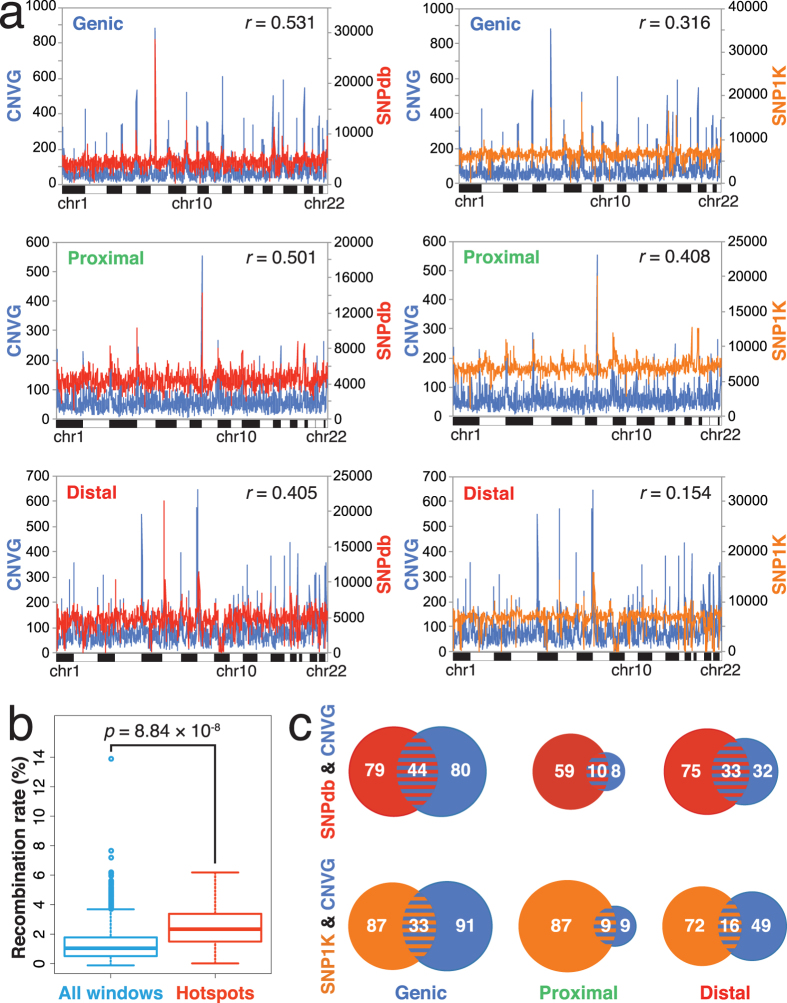
Co-localization of SNP and CNV hotspots. (**a**) Distributions of SNPdb, SNP1K and CNVG features in windows of Genic, Proximal and Distal zones. SNPdb (from dbSNP build 138) features are shown in red in the left hand side panels, SNP1K (from SNP1K phase 1, release v3) in orange in the right hand side panels, and the germline CNVG breakpoints in blue. Eliminating gaps in the genome devoid of known sequence, each of the three kinds of features are spread out at a fairly even basal occurrence level over the 500-kb non-overlapping windows along the lengths of the 22 autosomes on the x-axis. However, there are distinct red, orange and blue spikes indicative of sharply enhanced, or hotspot, occurrences. Some of the spikes are double red-blue or orange-blue hotspots where top-5% occurrence levels in both SNPs and CNVs are detected within the same 500-kb window. (**b**) Recombination rates for all 500-kb windows in the genome (left) and in SNP-CNV double hotspot windows with triple top-5% levels with respect to SNPdb, SNP1K and CNVG (right). The rates, obtained from RecH based on HapMap data, are compared using two-tailed t-test. (**c**) Venn diagrams of hotspots (viz. with top-5% level) in SNPdb (red area, >6,250 per window), SNP1K (orange area, >8,450 per window) and CNVG (blue area, >200 per window) in the three types of genomic zones. In each diagram, each segment size is proportional to the number of hotspots in the segment as indicated in the segment, e.g. top left diagram shows the presence of 79 + 44 = 123 SNPdb hotspots, 80 + 44 = 124 CNVG hotspots, and 44 double SNPdb-CNVG hotspots in the Genic zones of the 22 autosomes. The locations of individual SNPdb, SNP1K and CNVG hotspots, and their overlapped hotspots, are given in Supplementary Tables S6.

Although CNVG displays distinctly blue squares with both SNPdb and SNP1K, CNVG forms numerous blue squares whereas SNP1K and SNPdb form more red than blue squares with features in the Genic zones (Fig. 1a). In view of this, the chromosomal distributions of CNVG breakpoint, SNP1K and SNPdb occurrences (shown on y-axes, Fig. 7a) were compared using 500-kb windows. The distributions of CNVG and SNP (either SNPdb in the left-hand panels or SNP1K in the right-hand panels) are similar in character: while vast numbers of windows in the twenty-two autosomes (shown on x-axes) exhibit modest basal levels of SNPs or CNVs, some windows appear as sharp peaks with levels of SNPs or CNVs falling into the top-5% amongst all 500-kb windows, representing thereby hotspots in SNP or CNV occurrences. Furthermore, some of these hotspot windows are in fact double-hotspots displaying top-5% occurrences of SNPs as well as CNVs. Notably, the average recombination rate of 2.444 ± 1.430 based on RecH for the 54 CNV-SNP double-hotspot windows that register triple top-5% levels in SNPdb, SNP1K and CNVG features is significantly higher than the average recombination rate of 1.237 ± 0.984 for the 5,414 windows analyzed (*p*-value = 8.84 × 10^−8^, two-tailed t-test, Fig. 7b).

Figure 7c shows the numbers of windows that are CNVG, SNPdb or SNP1K hotspots, and SNPdb-CNVG or SNP1K-CNVG double hotspots in the three types of zones. Although the numbers of SNPdb or SNP1K hotspots do not vary greatly between the three types of zones, the 124 CNVG hotspots detected in Genic-zone windows exceed considerably the 18 CNVG hotspots detected in Proximal-zone windows.

**Figure 8:**
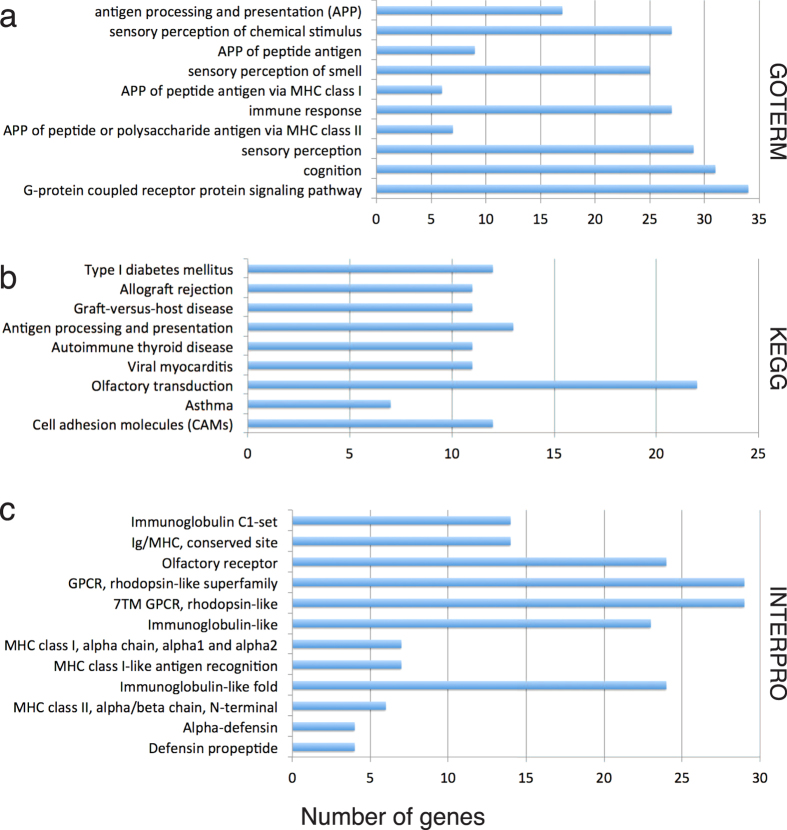
Enrichment analysis of genes in windows with triple top-5% levels of SNPdb, SNP1K and CNVG features. The 802 genes in the 54 hotspot 500-kb windows are functionally annotated using DAVID Bioinformatics Resources based on (**a**) GOTERM, (**b**) KEGG, (**c**) INTERPRO databases. Only those gene-groups yielding <0.05 Bonferroni corrected *p*-value, Benjamini corrected *p*-value as well as FDR *q*-value are considered to be significantly gene-enriched. The bars in each of (**a**–**c**) show the number of genes enriched in various gene groups, and the different bars are arranged from top down in order of increasing Bonferroni corrected *p*-values.

In Fig. 7a, there are 91 double SNP-CNV windows that display a top-5% level of CNVG together with a top-5% level of at least one of SNPdb and SNP1K. Fifty-four of the double SNP-CNV windows in fact display triple top-5% levels of CNVG, SNPdb as well as SNP1K, and they contain among them the partial or complete sequences of 802 genes. Functional annotation of these 802 genes based on DAVID Bioinformatics Resources^28^,^29^ identified gene-groups in the GOTERM, KEGG and INTERPRO databases that are enriched with these annotated genes, showing in each instance *p*- or *q-*values of <0.05 after each of the Bonferroni, Benjamini and FDR corrections (Supplementary Table S6). These gene-groups are plotted in Fig. 8 in bar graphs showing the 4–34 annotated genes within each group. The identified gene-groups in GOTERM include immune response, sensory perception of chemical stimulus and smell, cognition, and G-protein coupled receptor protein signaling pathway. In addition, the identified gene-groups in KEGG and INTERPRO include Type I diabetes mellitus, viral myocarditis, asthma, cell adhesion molecules, rhodopsin-like superfamily and alpha-defensin.

## Discussion

A large majority of the forty-two genomic features analyzed in Fig. 1a show measurable positive or negative co-localizations with other features. For most of the pairwise co-localizations, their co-localization coefficients are stable over window sizes between 50-kb and 2,000-kb. The main reason for any group of features to form blue squares with one another is that their densities or intensities co-vary non-randomly among different sequence windows. Accordingly a block of coherent blue squares formed by a group of features is indicative of their shared propensity to locate preferentially in the same target windows, thereby furnishing a basis for classifying such target windows into a distinct type of sequence zones. Consequently, although human genomic sequences are conventionally divided only between genic and intergenic regions, the three blocks of blue squares formed by Groups I, II and III features in Fig. 1a point to a fundamentally tripartite genome architecture consisting of three major types of sequence zones, viz. the Genic, Proximal and Distal zone windows. With regard to the red squares also, this three zonal-type architecture is reinforced by the all-red squares formed by L1 and ATrich with both Genic and Proximal zone features in Fig. 1a, thereby distinguishing the Distal zones they are enriched in from the Genic and Proximal zones. Likewise, the numerous red squares formed by L2, MIR and CID (the exceptionally high *r* value of which with TFBS signals their tight association) with a range of Genic zone features, as well as the much stronger red squares formed by CNVG with all four Proximal zone features compared to Genic zone features, accentuate the distinction between the Proximal and Genic zones.

The co-localizations between the marker features and functional features can furnish useful guides toward the selection of markers for phenotype-genotype associations. SNPs have been widely employed as markers in the CDCV search strategy^3^,^4^,^5^, but it has been pointed out that the utility of SNPs may be limited^6^,^7^,^8^. Accordingly, a CDRV approach has also been proposed based on rare structural variants such as CNVs^30^,^31^, which is supported by the blue squares formed by CNVG with various functional features in Genic zones, and machine learning-assisted analysis of recurrent germline CNVs as a basis for prediction of predisposition to cancer^23^,^26^. The finding of hotspot and basal SNPs, hotspot and basal CNVs, and double-hotspots with elevated levels of both SNPs and CNVs suggest that the enhanced diversity observed at such hotspot windows may not be readily accountable by genetic drift alone, and thus point to ongoing positive selection and recombination^32^. This is confirmed by the significantly increased recombination rates found in the SNPdb-SNP1K-CNVG triply top-5% windows relative to genome-wide basal rates, with *p*-value = 8.84 × 10^−8^ based on two-tailed t-test (Fig. 7b).

Since SNP hotspots, CNV hotspots and especially double SNP-CNV hotspots could signal the presence of genomic instability in a region, there is a possibility that such regions might be related with common diseases and complex traits. Accordingly, besides weighing between the relative merits of SNPs and CNVs as the basis or CDCV versus CDRV search strategies, it could be efficient to combine the usages of SNP and CNV and search for disease-genotype or complex phenotype-genotype associations employing a CDJV strategy based on the analysis of SNP and CNV hotspots. Implementation of CDJV can proceed by focusing searches on SNP-CNV double hotspots, SNP hotspots and CNV hotspots, which are listed in Supplementary Tables S6 and will grow with continued research on SNPs and CNVs. To expand coverage, the cut-off for hotspot SNP or CNV levels can be lowered from top-5% to top-10% levels, top-15% etc. Other marker features such as MSTs and CIDs may also be added to CDJV.

As indicated by Fig. 8a–c, the genes in SNP-CNV double hotspot windows with top-5% levels of SNPdb, SNP1K as well as CNVG occurrences are enriched in gene-groups related to immune response, alpha-defensin, sensory perception including olfactory and rhodopsin-like systems, Type I diabetes mellitus, cognition and G-protein coupled receptor protein signaling pathway. Diversity in immune response and alpha-defensin is understandable in view of the constant exposure of humans to evolving pathogens exemplified by novel or drug resistant bacteria and viruses. Diversity in sensory perception including the olfactory and rhodopsin-like systems might have accompanied adaptive evolution of the human lineage in a transition from arboreal life to life on the plains. Type I diabetes mellitus diversity could be related to change of human diet^33^, and G-protein signalling pathway diversity could be associated with blood pressure control and metabolic diseases in general^34^,^35^. Cognition diversity is likely due to the development of cognition as a human trait, exemplified by the active recombination and positive selection in the AluYi6 region of *GABRB2* found by us to be associated with not only schizophrenia and bipolar disorder but also social cognition^25^,^36^,^37^,^38^.

In contrast to the formation by CNVG of blue squares with some features in Genic zones, but red squares with the four features in Proximal zones, CNVT forms blue squares with all the features in Proximal zones and a widest range of features in Genic zones (Fig. 1a). The expanded positive co-localizations of CNVT compared to CNVG testify to the widespread infiltration of sequences in the Proximal and Genic zones by cancer somatic CNVs. The highest *r*-values formed by CNVT for 500-kb windows are the ones toward AluJ (0.57), AluS (0.57), FLAM (0.55), FAIRE (0.55), EXPS (0.54), MeMRE (0.54), REG (0.53), CpGi (0.0.41) and AluY (0.40) (Fig. 5, Supplementary Table S2). Given the known capacity of Alu elements to enhance recombination^12^,^13^,^39^,^40^, the possibility that CNVT infiltrations might be facilitated by Alu elements cannot be excluded. On the other hand, the low infiltration of Distal sequences by CNVT, indicated by the red squares formed by CNVT with NAS-, L1 and ATrich, is consistent with the low-recombination character of Distal zones shown by the all-red squares formed by features in these zones with RecH and RecD.

The three Alu elements form blue squares with high *r*-values among themselves: 0.89 between AluJ and AluS, 0.77 between AluS and AluY, and 0.64 between AluJ and AluY. These high *r* values, together with the fact that the lowest *r* value amongst them is exhibited by the AluJ-AluY pair with the largest age-gap between them, suggest that different regions of the human genome might have opened up to bursts of Alu-element attacks during its evolutionary history^13^,^41^. Time of entry also could be a factor in the strong positive co-localization between L2 and MIR (*r* value = 0.67, Fig. 5); it has been suggested that the transposition of both of these elements ceased before the primate-rodent split, and both of them are also commonly found in DNA segments conserved between the mouse and human genomes^19^,^42^.

Transcription factors (TFs) recognize TFBS sites, and play pivotal roles as determinants of cellular phenotypes in multicellular eukaryotes^43^,^44^. CIDs consist of functionally unannotated conserved indels in non-genic DNAs. While they are indicated to be functional by virtue of their conservation between human (hg19), mouse (mm8) and dog (camFam2) genomes, their exact function poses a challenge for the post-genomic era that has been recalcitrant to both *in silico* and functional approaches^16^,^42^,^45^ even though evidence has been accumulating for the involvement of CIDs in RNA processing or regulation of transcription and development^45^,^46^. In this regard, it is striking that the co-localization coefficient between the ~2.5 M CIDs and ~5.5 M TFBS is 0.91, the highest among the 861 pairwise co-localizations analyzed with an asymptotic *P* value of «10^−16^, suggesting tightly linked positionings of CIDs and TFBSs in the genome (Supplementary Table S2.7). The Circos diagrams also render evident the extraordinary parallelism between the distributions of CIDs and TFBSs (Fig. 2a,b, Supplementary Fig. S2). Furthermore, the strongest red squares formed by CNVG in Fig. 1a are those with the four Proximal zone features of L2 (*r* = −0.34), MIR (*r* = −0.22), CID (*r* = −0.24) and TFBS (*r* = −0.20) (Fig. 5), indicating that Proximal zones are highly sensitive to moderate or large distance perturbations. Together, these quantitative evidence based on genome-wide feature occurrences suggest that CIDs could participate as essential components of TFBS-CID arrays, where CIDs fulfil the important function of calibrated spacers setting the evolution-optimized distances in or surrounding such arrays, including the distances between different TFBS sites and/or between TF-TFBS complexes and their target promoters, genes or other regulatory elements. This is supported by the diverse findings of highly conserved non-coding sequences between humans and *Fugu* located principally around human genes involved with regulation of development and transcription factors^45^, ultra-conserved segments between human, rat and mouse genomes, including numerous CIDs, that are closely associated with genes participating in RNA processing or regulation of transcription and development^46^, and the conservation of spacings between conserved non-coding sequences in *Drosophila*^16^,^47^. Moreover, the inclusion of MIRs in the Proximal zones, and the finding of MIR-derived enhancers as a rich source of TFBSs^48^, increase the possibility of the TFBS-CID arrays being the focal functional units in Proximal zones.

In conclusion, pairwise co-localizations between various genomic features, by quantifying the correlations between the locations of the features, have revealed a three zonal-type human genome architecture. Their utility for genomic medicine is exemplified by the surprising finding of double hotspots of SNPs and CNVs, enabling a CDJV search strategy for disease-genotype and complex phenotype-genotype associations, and evidence for dissimilarity between CNVG and CNVT with respect to their co-localization profiles. Their close relationships with genome evolution are illustrated by the strong positive co-localizations between the AluJ, AluS and AluY elements, and between the L2 and MIR elements. Their importance to genomic function is demonstrated by the highest co-localization coefficient identified for the CID-TFBS pair among all pairwise co-localizations analyzed, pointing to a space-determinant functional role for CIDs. Feature co-localizations therefore provide an efficient approach for deriving from the extensive feature databases deepened insights into the human genome.

## Methods

### Data sources

Raw data (*.txt) and schema (*.sql) of the forty-two genomic features analyzed in this study were retrieved from UCSC GoldenPath FTP site (ftp://hgdownload.cse.ucsc.edu/goldenPath/hg19/database)^49^,^50^, except for CNVT from the SNP6 copy number analysis (GISTIC2; version analyses_2014_10_17) of Board GDAC Firehose^51^. Supplementary Table S1 lists the details of various features and their sources, including their track names in UCSC and DOI references to Firehose GDAC analyses.

### Feature quantitation in fixed-size windows

The sequences of each autosome were binned into successive fixed-size 50, 200, 500 or 2,000-kb windows starting from residue-1, e.g. chromosome 1 with a length of 249 Mb in human reference genome hg19 yielded 498 windows of 500-kb. Windows that overlapped with UCSC “gap” tracks, including gap regions (long stretches of Ns), telomeres, centromeres, heterochromatin regions, and regions with no available sequence information in the reference genome, were removed from further analysis. For the purpose of correlating between the levels of any two features in fixed-size windows, two kinds of features were treated differently. For features where the numbers of bases were simply counted, their levels were expressed in ‘Density’, e.g. for the level of L1 in any 500-kb window:


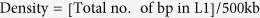


On the other hand, for features where the numbers of bases were counted, and each feature was also assessed by a numerical score, their levels were expressed in ‘Intensity’, e.g. for the level of TFBS in any 500-kb window:


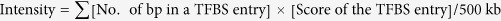


On this basis, pairwise correlation was performed between the Density or Intensity of one feature and the Density or Intensity of a second feature. The features expressed in Density and those in Intensity are listed in Supplementary Table S1.

### Feature co-localization analysis

For any pair of features, input of their paired levels (i.e. ‘Density’ or ‘Intensity’) in all 5,414 windows (Supplementary Table S1) into the “rcorr” function “pearson” type in Hmisc package under R environment yielded Pearson correlation coefficient *r* and the asymptotic *P*-value between the pair of features, whereas input into the “rcorr” function “spearman” type yielded Spearman correlation coefficient.

### Genes located in CNV-SNP co-localization hotspots

Windows with triple top-5% levels of SNPdb, SNP1K and CNVG features were uploaded to BioMart of Ensembl database to generate a list of their gene contents; any gene is regarded as being part of the content of a window if any portion of its sequence falls within the window. This gene list was next uploaded to DAVID Bioinformatics Resources 6.7^28^,^29^, using ‘Functional Annotation Tool’ to obtain the enrichments of the genes in the gene-groups including gene-terms, functional groups and pathways indicated in Supplementary Table S6.5. Only those gene-groups yielding <0.05 Bonferroni corrected *p*-value, Benjamini corrected *p*-value as well as FDR *q*-value were regarded to be significantly enriched in the genes in the gene-list.

### Software employed in data processing and visualization

Data processing tasks were carried out using custom Python codes, except that tasks requiring text processing (regular expression functionalities) were implemented in Perl codes, and C/C++ language was employed where processing speed was otherwise too slow. All figures were drawn using the ggplot2 and lattice packages under R environment, except for Fig. 2 and Supplementary Fig. S2 which were drawn using the Circos program^52^.

## Additional Information

**How to cite this article**: Ng, S.-K. *et al.* Feature co-localization landscape of the human genome. *Sci. Rep.* **6**, 20650; doi: 10.1038/srep20650 (2016).

## Supplementary Material

Supplementary Information

Supplementary Table S1

Supplementary Table S2

Supplementary Table S3

Supplementary Table S4

Supplementary Table S5

Supplementary Table S6
