# Supplementary material for: Feature co-localization landscape of the human genome
Source: Sci Rep. 2016 Feb 8;6:20650. doi: 10.1038/srep20650 (PMC4745063; doi:10.1038/srep20650)
Supplement: Supplementary Information [file srep20650-s1.pdf]

Feature co-localization landscape of the human genome

Siu-Kin Ng*, Taobo Hu*, Xi Long, Cheuk-Hin Chan, Shui-Ying Tsang & Hong Xue

Division of Life Science, Applied Genomics Center and Center for Statistical Science,

Hong Kong University of Science and Technology, Clear Water Bay, Hong Kong

*These authors contributed equally to this work

Correspondence to be addressed to:

Hong Xue

Division of Life Science

Hong Kong University of Science and Technology

Clear Water Bay, Hong Kong

hxue@ust.hk

Supplementary information

Supplementary Table S1. Information on forty-two genomic features analyzed for pairwise co-localizations.

Supplementary Table S2. Pearson correlation coefficients and asymptotic *P* values, and Spearman correlation coefficients, between different pairs of features. **S2.1** Pearson correlation coefficients between different feature-pairs based on 50-kb windows. **S2.2** Asymptotic *P*-values of Pearson correlations between different feature-pairs based on 50-kb windows. **S2.3** Pearson correlation coefficients between different feature-pairs based on 200-kb windows. **S2.4** Asymptotic *P*-values of Pearson correlations between different feature-pairs based on 200-kb windows. **S2.5** Pearson correlation coefficients between different feature-pairs based on 500-kb windows. **S2.6** Asymptotic *P*-values of Pearson correlations between different feature-pairs based on 500-kb windows. **S2.7** Asymptotic *P*-values of Pearson correlations between different feature-pairs based on 500-kb windows, as employed in Figure 5, with t values. **S2.8** Pearson correlation coefficients between different feature-pairs based on 2,000-kb windows. **S2.9** Asymptotic *P*-values of Pearson correlations between different feature-pairs based on 2,000-kb windows. **S2.10** Spearman correlation coefficients between different feature-pairs based on 50-kb windows. **S2.11** Spearman correlation coefficients between different feature-pairs based on 200-kb windows. **S2.12** Spearman correlation coefficients between different feature-pairs based on 500-kb windows. **S2.13** Spearman correlation coefficients between different feature-pairs based on 2,000-kb windows. **S2.14** Average

difference of Pearson correlation coefficients for various window sizes. **S2.15** Average difference of Pearson and Spearman correlation coefficients for various window sizes.

Supplementary Table S3. Length distributions and abundances of various types of DSVs.

Supplementary Table S4. Feature compositions of sequence windows. **S4.1** Compositions of 5,414 500-kb windows and their classification into Genic, Proximal and Distal zones based on the feature-ratios method described in Supplementary Figure S4. **S4.2** Compositions of 50-kb windows in the 59.50-62.50 Mb segment of chromosome 5.

Supplementary Table S5. Fractional distributions of different features among Genic, Proximal and Distal zones.

Supplementary Table S6. Hotspots in SNPdb, SNP1K and CNVG features. **S6.1** Hotspots in Genic zones. **S6.2** Hotspots in Proximal zones. **S6.3** Hotspots in Distal zones. **S6.4** Genes within hotspot windows. **S6.5** Functional annotation of genes within hotspot windows using DAVID. **S6.6** Distribution of functionally annotated genes among the three types of sequence zones.

Supplementary Figure S1. Heat maps of pairwise Pearson correlation coefficients (lower triangle) and Spearman correlation coefficients (upper triangle) between forty-two genomic features in (**a**) 50-kb, (**b**) 200-kb, (**c**) 500-kb and (**d**) 2,000-kb windows in twenty-two

autosomes.

Supplementary Figure S2. Distribution of different genomic features on twenty autosomes. Circos diagrams show from outside inwards positions on chromosome in Mb, and abundances (Supplementary Table S4) of GENE (blue), L1 (red), Alu viz. sum of AluJ, AluS and AluY (green), TFBS (orange), CID (purple) and MIR (yellow) in 500-kb windows expressed in separate linear scales.

Supplementary Figure S3. Parameters of prominent co-localizations. Correlation coefficient *r* values and asymptotic *P* values are based on (**a**) 50-kb, (**b**) 200-kb and (**c**) 2,000-kb windows. See Figure 5 for explanations.

Supplementary Figure S4. Partition of genomic sequences among different sequence zones based on feature ratios. According to Figure 1a, L1 enrichment is a characteristic of Group III windows, whereas TFBS enrichment is a characteristic of Group I and Group II windows. As well, MIR forms stronger correlations with Group I than Group II features whereas Alu elements form stronger correlations with Group II than Group I features. Accordingly, as first approximation, indentations in the L1/TFBS and Alu/MIR distribution profiles can furnish plausible landmarks for separating the Genic, Proximal and Distal zones: (**a**) Distribution of 500-kb windows with different basepair ratios between the L1 and TFBS features. Arrow separates plausible Distal windows with L1/TFBS basepair ratio $\geq 1.05 \times 10^4$ and Genic + Proximal windows with L1/TFBS basepair ratio $< 1.05 \times 10^4$. (**b**) Distribution of Genic +

Proximal windows with different basepair ratios between Alu (representing the sum of AluY, AluS, AluJ and FLAM) and MIR. Arrow separates plausible Genic windows with Alu/MIR basepair ratio ≥ 2.37 and Proximal windows < 2.37. On this basis, the 5,414 non-gap 500-kb windows in the 22 autosomes consist of 45.1% Genic zones (comprising Group II features), 31.1% Proximal zones (comprising Group I features) and 23.8% Distal zones (comprising Group III features).

Supplementary Figure S5. Distribution of SNPs among three types of architectural zones. The proportions of Genic, Proximal and Distal zones in human genome (**a**); proportions of SNPs in dbSNP database (**b**), in the subset of dbSNP database with clinical significance (**c**), in ClinVar (CLV) (**d**), and employed in the Affymetrix 6.0 chips (**e**); and complex phenotype-associated SNPs discovered by GWAS (**f**) in Genic zones (blue), Proximal zones (green) and Distal zones (red). The number of SNPs and their percentile proportion are shown inside each of the zones.

Supplementary Figure S6. Enrichment analysis of genes in windows with triple top-5% levels in SNPdb, SNP1K and CNVG. The 802 genes in the 54 such windows are functionally annotated using DAVID Bioinformatics Resources based on GOTERM, KEGG and INTERPRO database classifications. The significant gene annotations that yielded (**a**) Bonferroni corrected p-value < 0.05, (**b**) Benjamini corrected p-value < 0.05 and (**c**) FDR corrected q-value < 0.05 are shown. Each bar graph indicates the –log [p or q value].

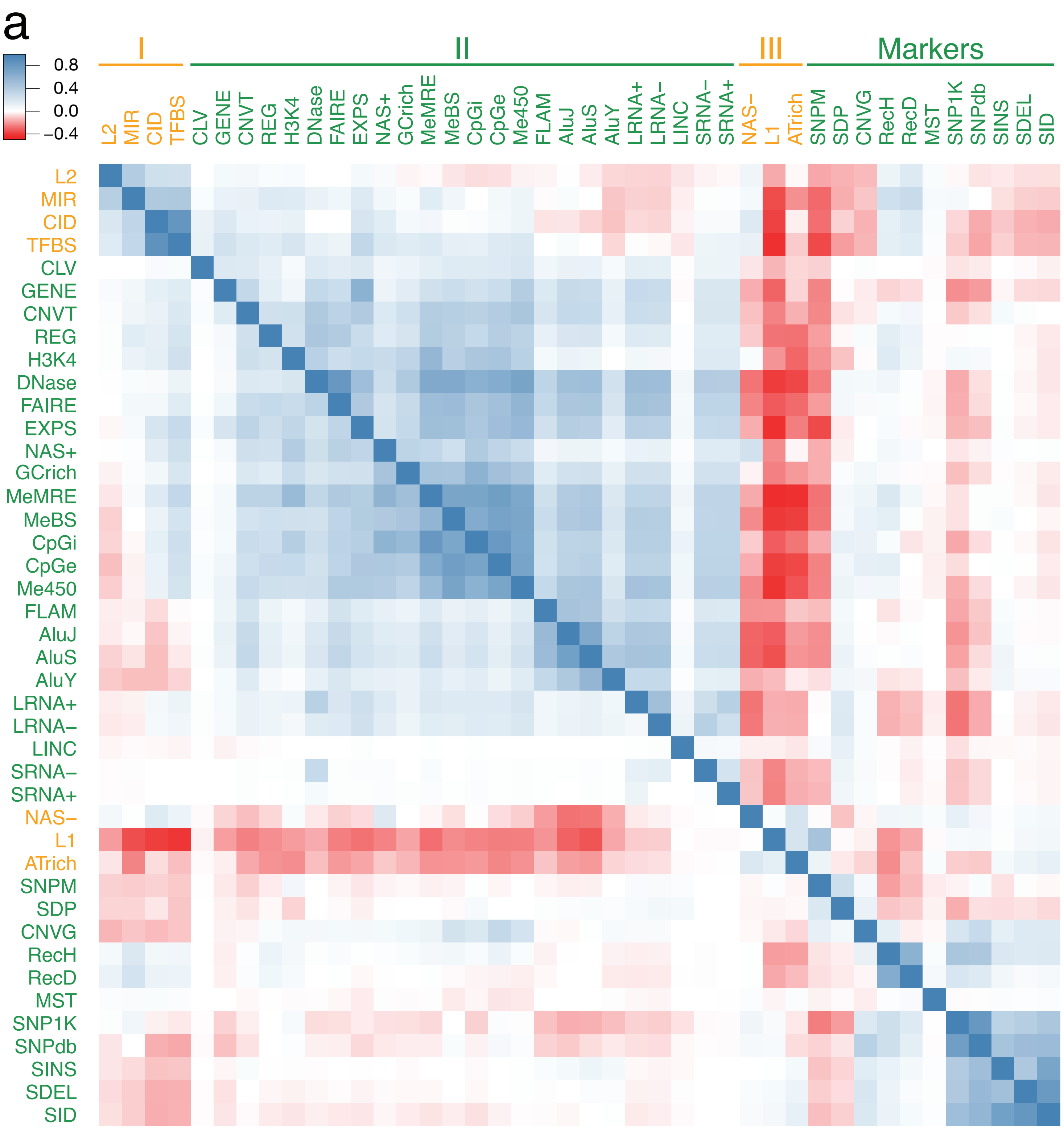

Supplementary Figure S1

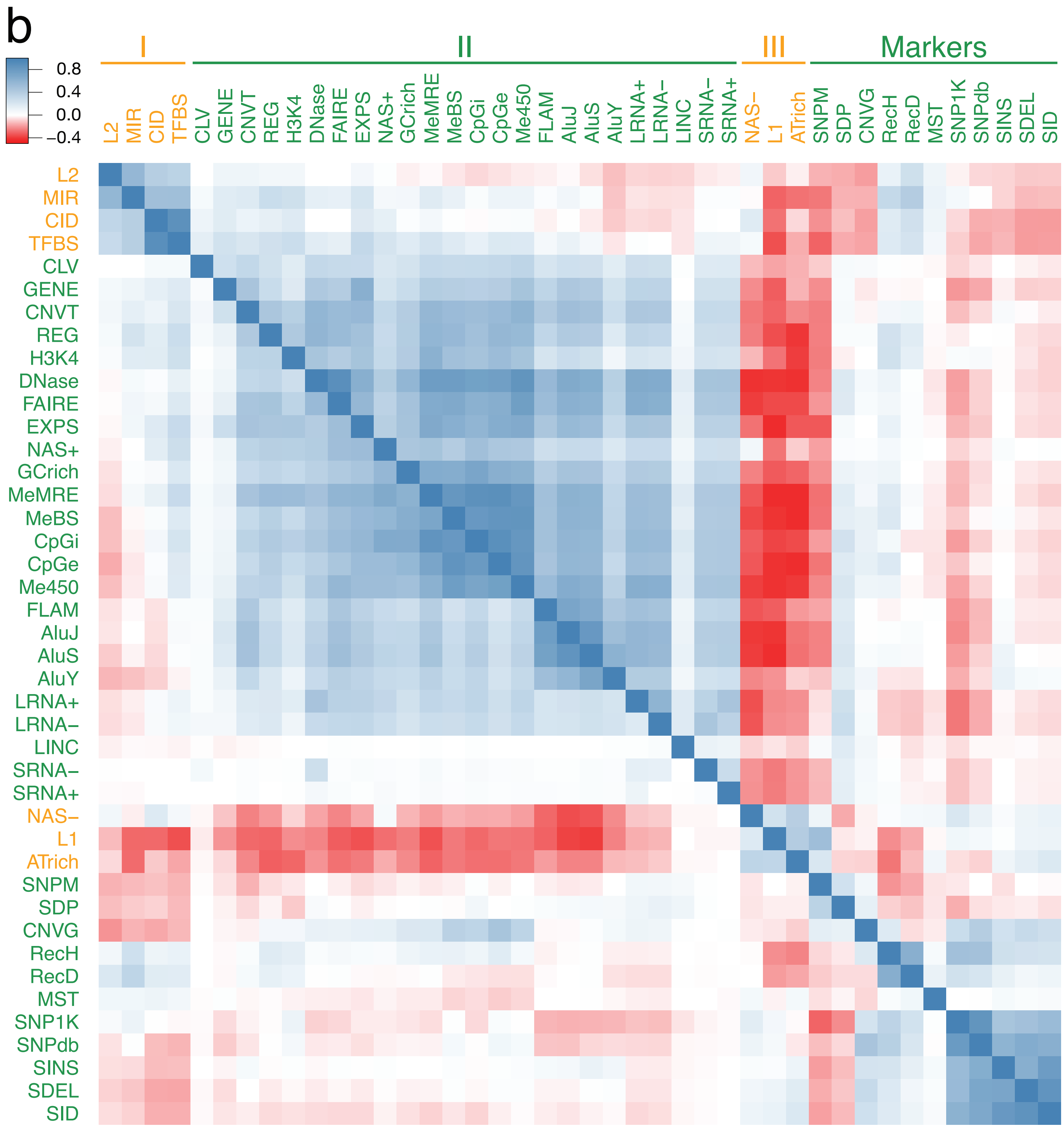

Supplementary Figure S1

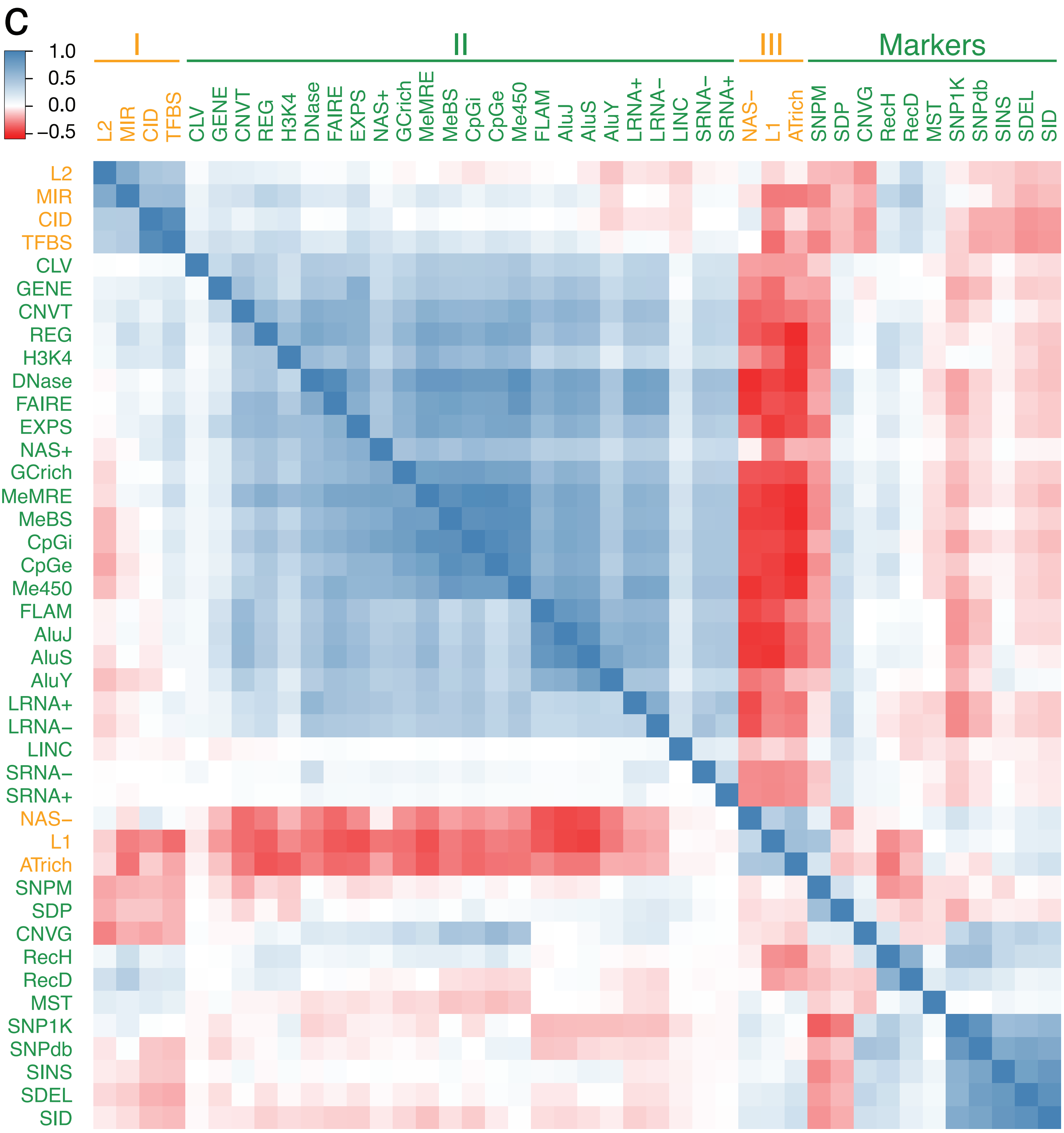

Supplementary Figure S1

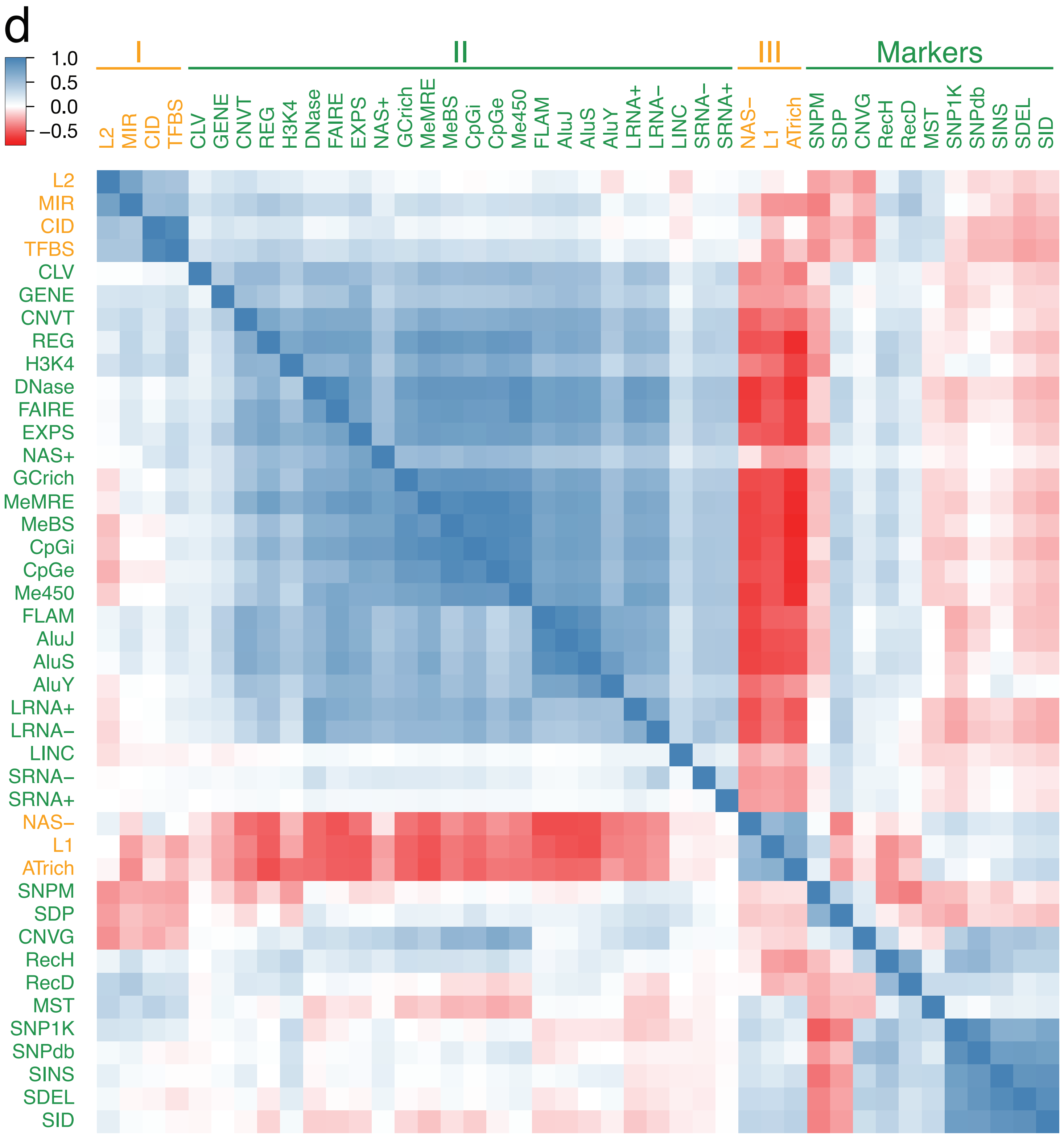

Supplementary Figure S1

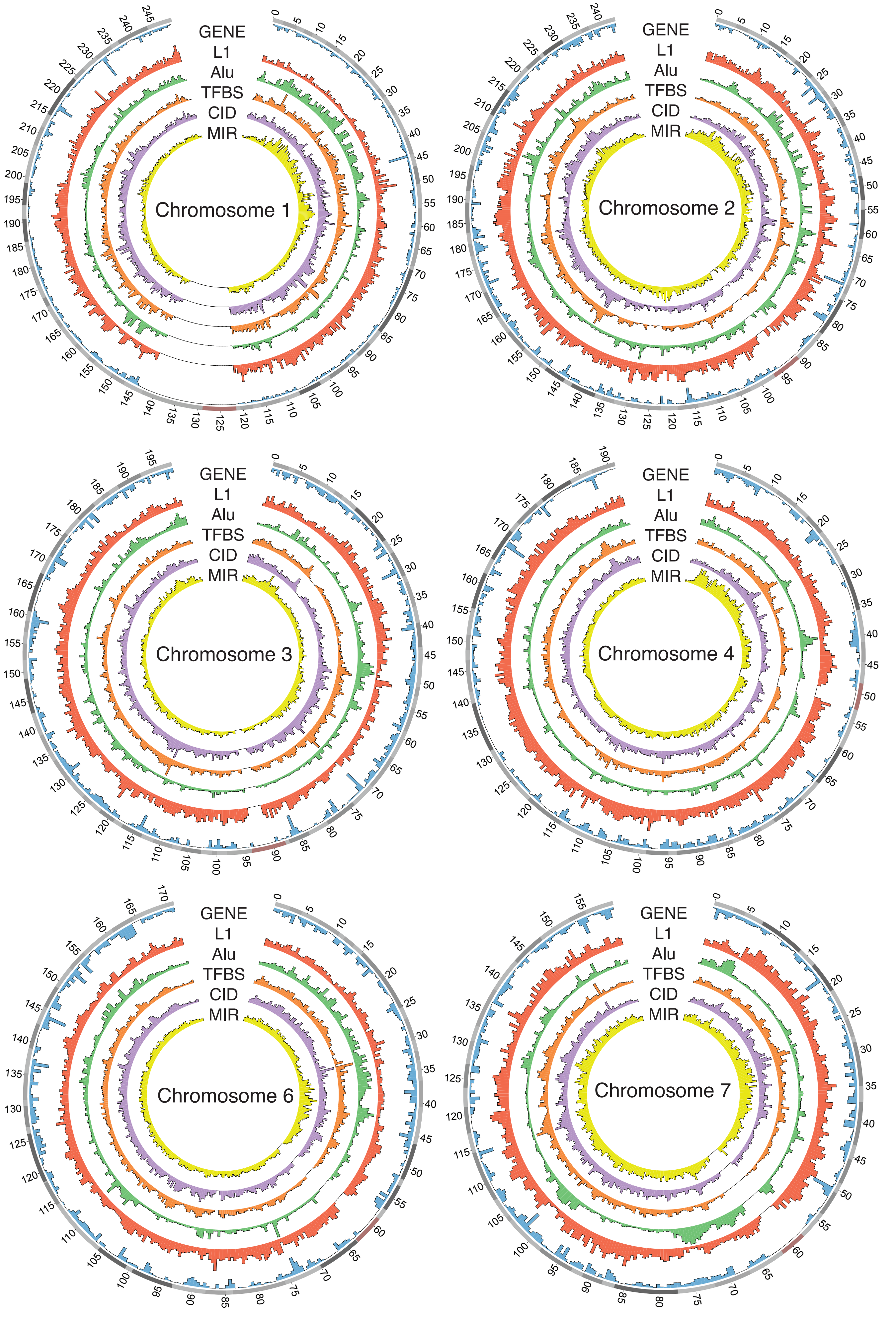

Supplementary Figure S2

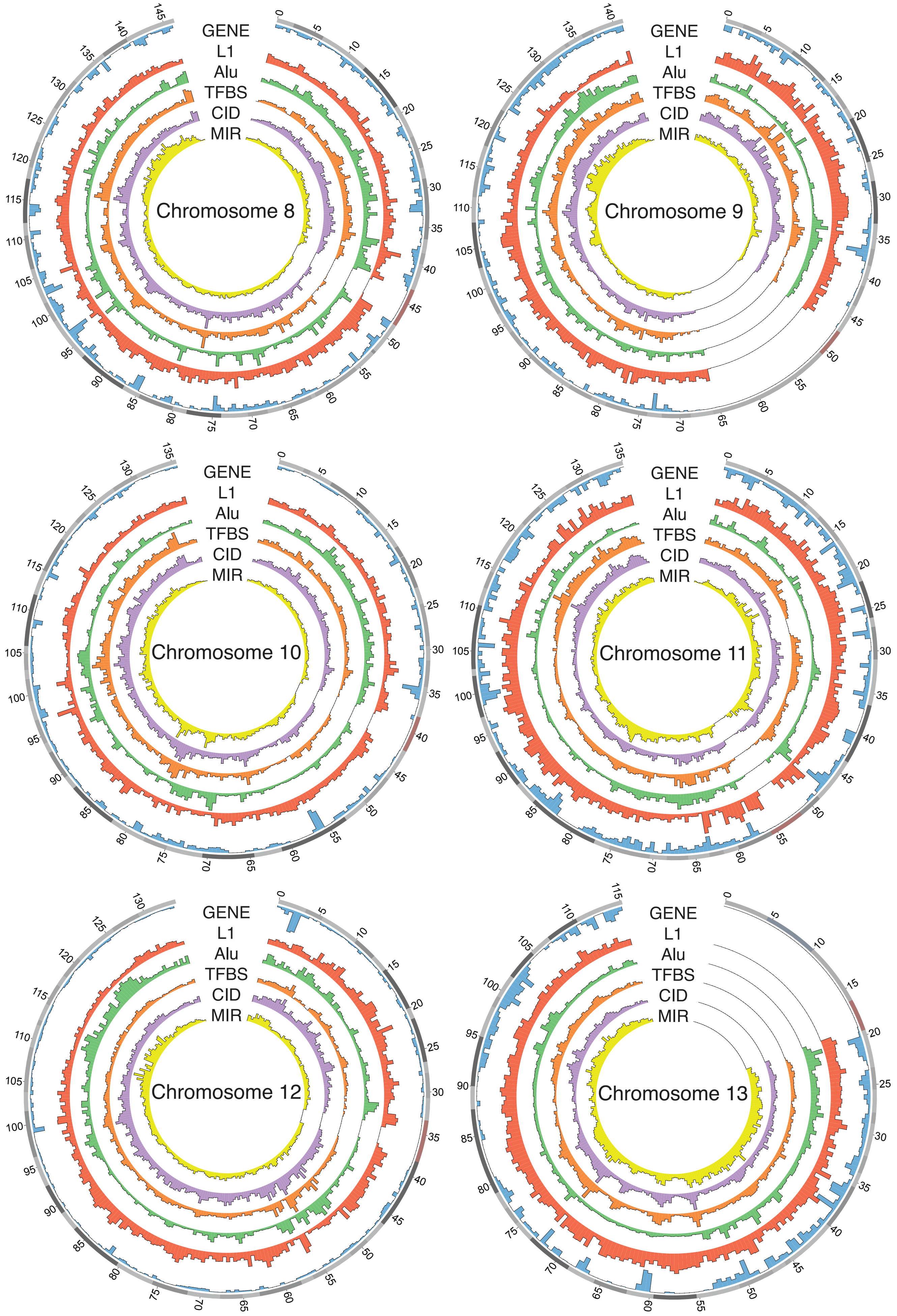

Supplementary Figure S2

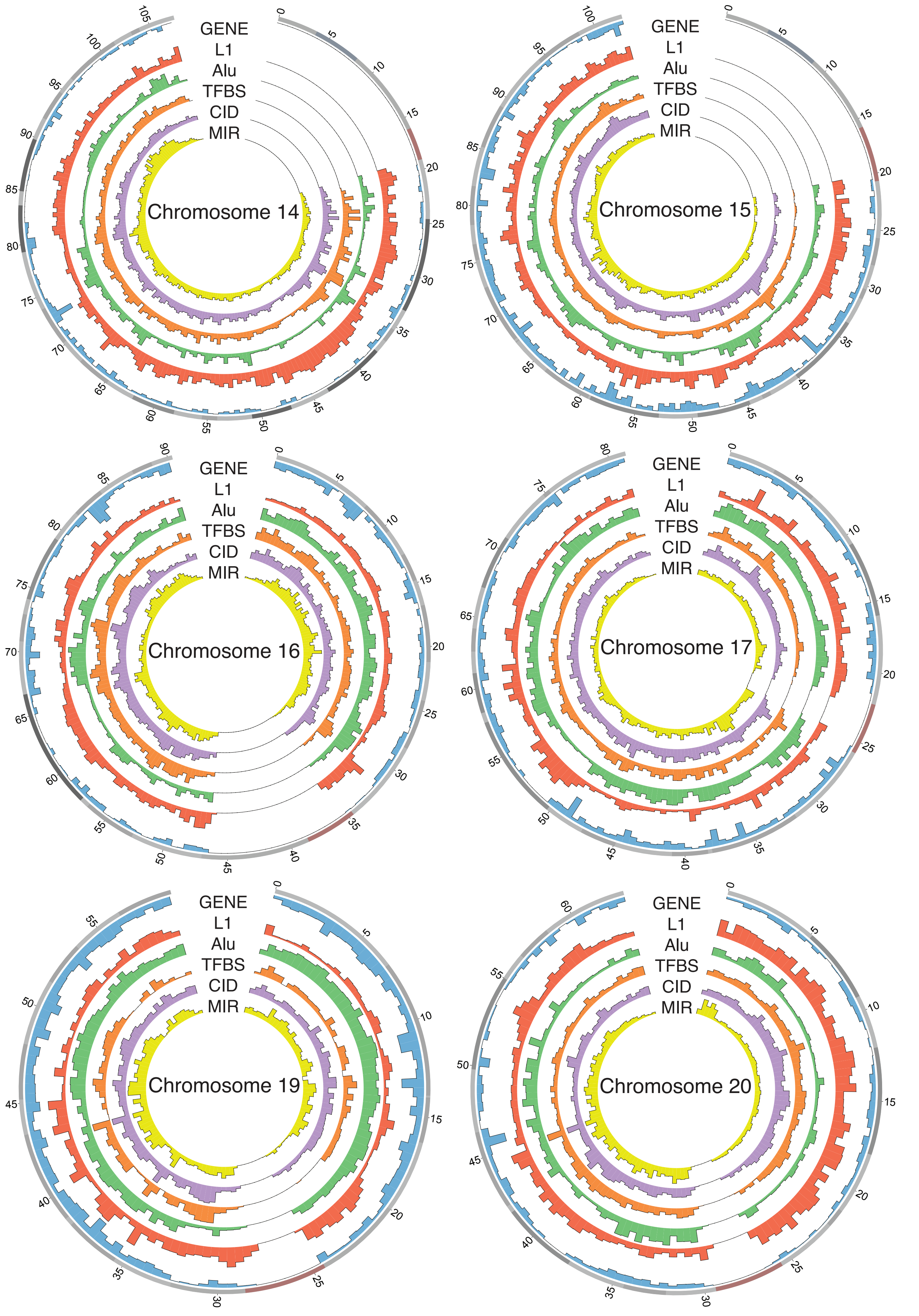

Supplementary Figure S2

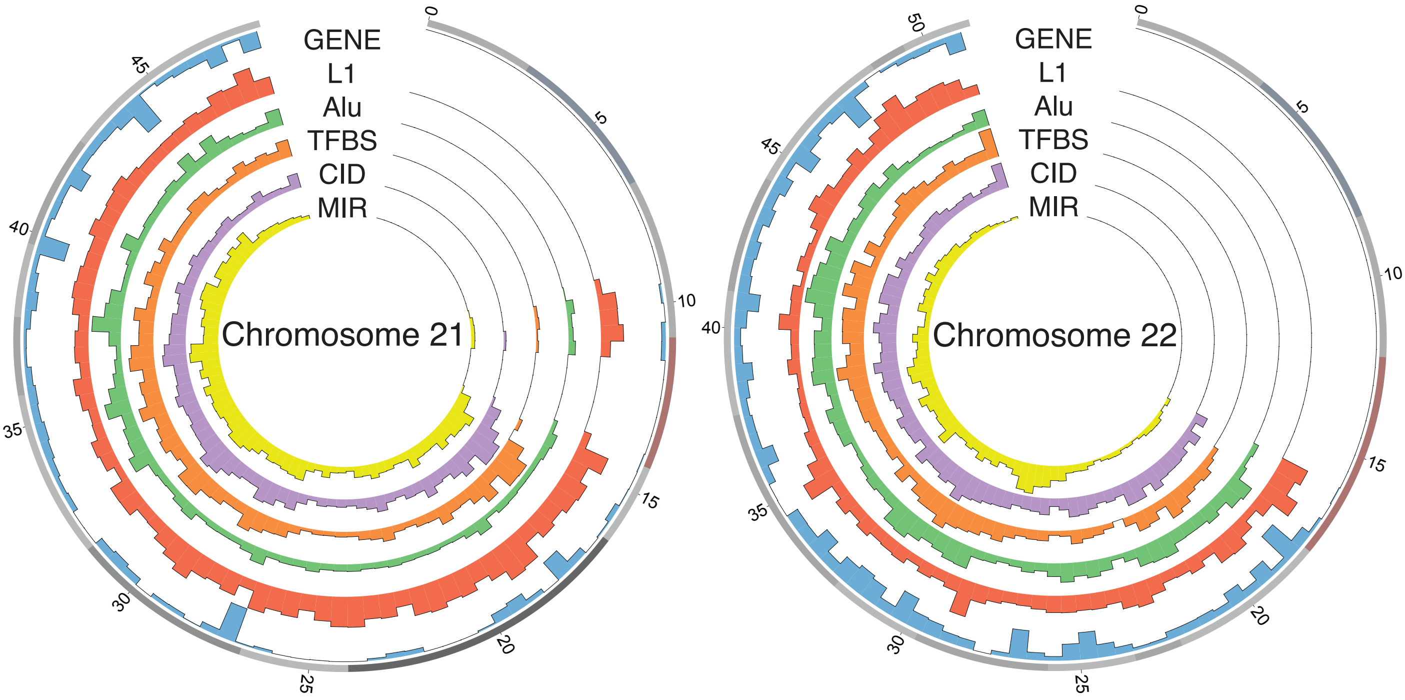

Supplementary Figure S2

a

L2	0.38	0.20	0.18	0.00064	0.047	0.031	0.023	−0.083	−0.04	−0.087	−0.10	−0.19	−0.081	−0.14	0.064	0.11	−0.049
0.00	MIR	0.33	0.33	0.0008	0.079	0.093	0.16	−0.015	−0.015	−0.052	−0.13	−0.35	−0.082	−0.11	0.19	0.23	0.0086
0.00	0.00	CID	0.87	0.047	0.15	0.12	0.14	0.15	−0.11	−0.13	−0.13	−0.39	−0.048	−0.13	0.08	0.11	−0.15
0.00	0.00	0.00	TFBS	0.042	0.17	0.20	0.22	0.27	−0.024	−0.042	−0.082	−0.41	−0.11	−0.11	0.088	0.11	−0.17
0.88	0.85	0.00	0.00	CLV	0.032	0.043	0.024	0.026	0.015	0.023	0.012	−0.026	−0.002	0.0049	−0.0013	−0.0011	−0.012
0.00	0.00	0.00	0.00	1.3e−13	GENE	0.17	0.062	0.058	0.13	0.12	0.059	−0.19	−0.032	−0.028	−0.035	−0.031	−0.12
1.4e−12	0.00	0.00	0.00	0.00	0.00	CNVT	0.25	0.29	0.31	0.32	0.17	−0.25	−0.052	−0.023	0.026	0.034	−0.055
1.0e−07	0.00	0.00	0.00	2.0e−08	0.00	0.00	REG	0.27	0.15	0.15	0.062	−0.22	−0.018	0.041	0.11	0.091	0.0057
0.00	0.00046	0.00	0.00	2.6e−09	0.00	0.00	0.00	CpGi	0.21	0.23	0.16	−0.24	0.055	0.20	0.053	−0.023	−0.027
0.00	0.00066	0.00	2.8e−08	0.00065	0.00	0.00	0.00	0.00	AluJ	0.75	0.44	−0.31	0.036	−0.018	−0.0035	0.0097	−0.10
0.00	0.00	0.00	0.00	1.5e−07	0.00	0.00	0.00	0.00	0.00	AluS	0.57	−0.34	0.042	0.015	0.011	0.012	−0.064
0.00	0.00	0.00	0.00	0.0043	0.00	0.00	0.00	0.00	0.00	0.00	AluY	−0.17	0.03	0.053	−0.028	−0.042	−0.043
0.00	0.00	0.00	0.00	1.2e−09	0.00	0.00	0.00	0.00	0.00	0.00	0.00	L1	−0.024	−0.044	−0.19	−0.17	0.053
0.00	0.00	0.00	0.00	0.65	1.5e−13	0.00	1.8e−05	0.00	0.00	0.00	5.7e−12	1.3e−08	SDP	0.077	−0.036	−0.042	−0.051
0.00	0.00	0.00	0.00	0.26	9.9e−11	7.5e−08	0.00	0.00	2.3e−05	0.00045	0.00	0.00	0.00	CNVG	0.13	−0.022	0.37
0.00	0.00	0.00	0.00	0.77	8.9e−16	2.0e−09	0.00	0.00	0.42	0.0089	7.9e−11	0.00	0.00	0.00	RecH	0.66	0.27
0.00	0.00	0.00	0.00	0.79	1.1e−12	2.7e−15	0.00	8.4e−08	0.024	0.0053	0.00	0.00	0.00	5.2e−07	0.00	RecD	0.15
0.00	0.047	0.00	0.00	0.0055	0.00	0.00	0.19	4.4e−10	0.00	0.00	0.00	0.00	0.00	0.00	0.00	0.00	SNPdb

Supplementary Figure S3

b

L2	0.58	0.34	0.30	0.0054	0.076	0.081	0.057	−0.15	−0.062	−0.12	−0.17	−0.16	−0.15	−0.25	0.083	0.20	−0.066
0.00	MIR	0.39	0.38	0.0039	0.098	0.14	0.24	−0.036	0.0084	−0.031	−0.14	−0.36	−0.12	−0.17	0.26	0.35	0.018
0.00	0.00	CID	0.90	0.058	0.15	0.11	0.16	0.079	−0.073	−0.088	−0.11	−0.35	−0.10	−0.20	0.11	0.17	−0.15
0.00	0.00	0.00	TFBS	0.058	0.18	0.21	0.28	0.23	0.049	0.027	−0.031	−0.42	−0.16	−0.16	0.12	0.16	−0.17
0.53	0.65	1.2e−11	1.4e−11	CLV	0.045	0.078	0.054	0.057	0.036	0.045	0.04	−0.05	0.00078	0.0079	0.0036	0.0018	−0.017
0.00	0.00	0.00	0.00	1.3e−07	GENE	0.25	0.12	0.096	0.20	0.18	0.12	−0.25	−0.035	−0.027	−0.015	−0.019	−0.12
0.00	0.00	0.00	0.00	0.00	0.00	CNVT	0.42	0.36	0.49	0.49	0.32	−0.35	−0.087	−0.037	0.042	0.061	−0.062
3.3e−11	0.00	0.00	0.00	3.8e−10	0.00	0.00	REG	0.42	0.31	0.32	0.20	−0.36	−0.03	0.077	0.17	0.14	−0.0055
0.00	2.8e−05	0.00	0.00	3.4e−11	0.00	0.00	0.00	CpGi	0.37	0.39	0.34	−0.35	0.082	0.34	0.095	−0.059	−0.0074
7.8e−13	0.33	0.00	9.7e−09	3.6e−05	0.00	0.00	0.00	0.00	AluJ	0.85	0.58	−0.45	0.055	−0.016	−0.0095	0.011	−0.14
0.00	0.00028	0.00	0.0017	2.2e−07	0.00	0.00	0.00	0.00	0.00	AluS	0.72	−0.47	0.065	0.028	0.013	0.015	−0.095
0.00	0.00	0.00	0.00028	2.6e−06	0.00	0.00	0.00	0.00	0.00	0.00	AluY	−0.29	0.063	0.075	−0.04	−0.067	−0.083
0.00	0.00	0.00	0.00	6.7e−09	0.00	0.00	0.00	0.00	0.00	0.00	0.00	L1	−0.042	−0.06	−0.26	−0.23	0.061
0.00	0.00	0.00	0.00	0.93	4.6e−05	0.00	0.00045	0.00	1.6e−10	3.1e−14	2.8e−13	1.2e−06	SDP	0.13	−0.055	−0.083	−0.09
0.00	0.00	0.00	0.00	0.36	0.0017	1.9e−05	0.00	0.00	0.07	0.0011	0.00	3.8e−12	0.00	CNVG	0.19	−0.081	0.46
0.00	0.00	0.00	0.00	0.67	0.075	8.6e−07	0.00	0.00	0.27	0.12	4.3e−06	0.00	2.0e−10	0.00	RecH	0.64	0.38
0.00	0.00	0.00	0.00	0.84	0.026	1.7e−12	0.00	9.9e−12	0.20	0.077	9.8e−15	0.00	0.00	0.00	0.00	RecD	0.21
1.2e−14	0.039	0.00	0.00	0.045	0.00	8.0e−13	0.52	0.39	0.00	0.00	0.00	1.4e−12	0.00	0.00	0.00	0.00	SNPdb

Supplementary Figure S3

C

L2	0.74	0.49	0.45	0.022	0.21	0.27	0.12	−0.18	0.088	0.027	−0.076	−0.006	−0.30	−0.35	0.10	0.35	0.052
0.00	MIR	0.43	0.45	0.02	0.23	0.32	0.34	−0.0033	0.21	0.18	0.019	−0.30	−0.19	−0.21	0.27	0.43	0.09
0.00	0.00	CID	0.94	0.078	0.22	0.20	0.20	0.006	0.087	0.061	0.0074	−0.16	−0.23	−0.26	0.11	0.25	−0.029
0.00	0.00	0.00	TFBS	0.099	0.26	0.33	0.37	0.17	0.21	0.19	0.097	−0.28	−0.24	−0.19	0.14	0.24	−0.053
0.42	0.45	0.0041	0.00025	CLV	0.10	0.19	0.15	0.15	0.13	0.14	0.14	−0.12	0.007	0.034	−0.0017	−0.026	−0.03
1.3e−15	0.00	4.4e−16	0.00	0.00024	GENE	0.39	0.30	0.23	0.39	0.35	0.26	−0.26	−0.029	0.015	0.10	0.081	−0.0061
0.00	0.00	7.8e−14	0.00	1.2e−12	0.00	CNVT	0.66	0.47	0.65	0.66	0.50	−0.39	−0.12	0.035	0.16	0.19	0.049
4.0e−06	0.00	3.4e−13	0.00	1.3e−08	0.00	0.00	REG	0.61	0.58	0.60	0.48	−0.52	0.015	0.17	0.27	0.18	0.019
6.4e−11	0.90	0.83	2.0e−10	5.7e−08	0.00	0.00	0.00	CpGi	0.53	0.57	0.57	−0.47	0.21	0.57	0.22	−0.094	0.072
0.0012	1.3e−15	0.0014	1.6e−15	3.2e−06	0.00	0.00	0.00	0.00	AluJ	0.92	0.71	−0.55	0.11	0.056	0.11	0.13	−0.065
0.31	5.4e−11	0.023	6.0e−12	2.5e−07	0.00	0.00	0.00	0.00	0.00	AluS	0.82	−0.57	0.13	0.13	0.16	0.14	−0.013
0.0049	0.49	0.79	0.00032	3.9e−07	0.00	0.00	0.00	0.00	0.00	0.00	AluY	−0.40	0.20	0.23	0.13	0.041	0.0039
0.82	0.00	4.2e−09	0.00	1.8e−05	0.00	0.00	0.00	0.00	0.00	0.00	0.00	L1	−0.15	−0.13	−0.30	−0.23	0.13
0.00	1.5e−12	0.00	0.00	0.80	0.28	0.00002	0.57	2.0e−15	6.5e−05	7.5e−07	3.9e−13	4.0e−08	SDP	0.25	−0.075	−0.17	−0.20
0.00	1.4e−14	0.00	3.3e−12	0.20	0.59	0.20	1.7e−10	0.00	0.04	8.5e−07	0.00	3.0e−06	0.00	CNVG	0.35	−0.17	0.52
0.00021	0.00	6.1e−05	9.3e−08	0.95	0.00011	1.4e−09	0.00	0.00	2.5e−05	3.5e−09	2.8e−06	0.00	0.0054	0.00	RecH	0.55	0.57
0.00	0.00	0.00	0.00	0.33	0.0029	1.0e−12	8.4e−12	0.00049	1.2e−06	1.4e−07	0.13	0.00	4.4e−10	4.0e−10	0.00	RecD	0.30
0.053	0.00089	0.29	0.051	0.26	0.82	0.073	0.48	0.0079	0.017	0.64	0.88	1.1e−06	1.6e−13	0.00	0.00	0.00	SNPdb

Supplementary Figure S3

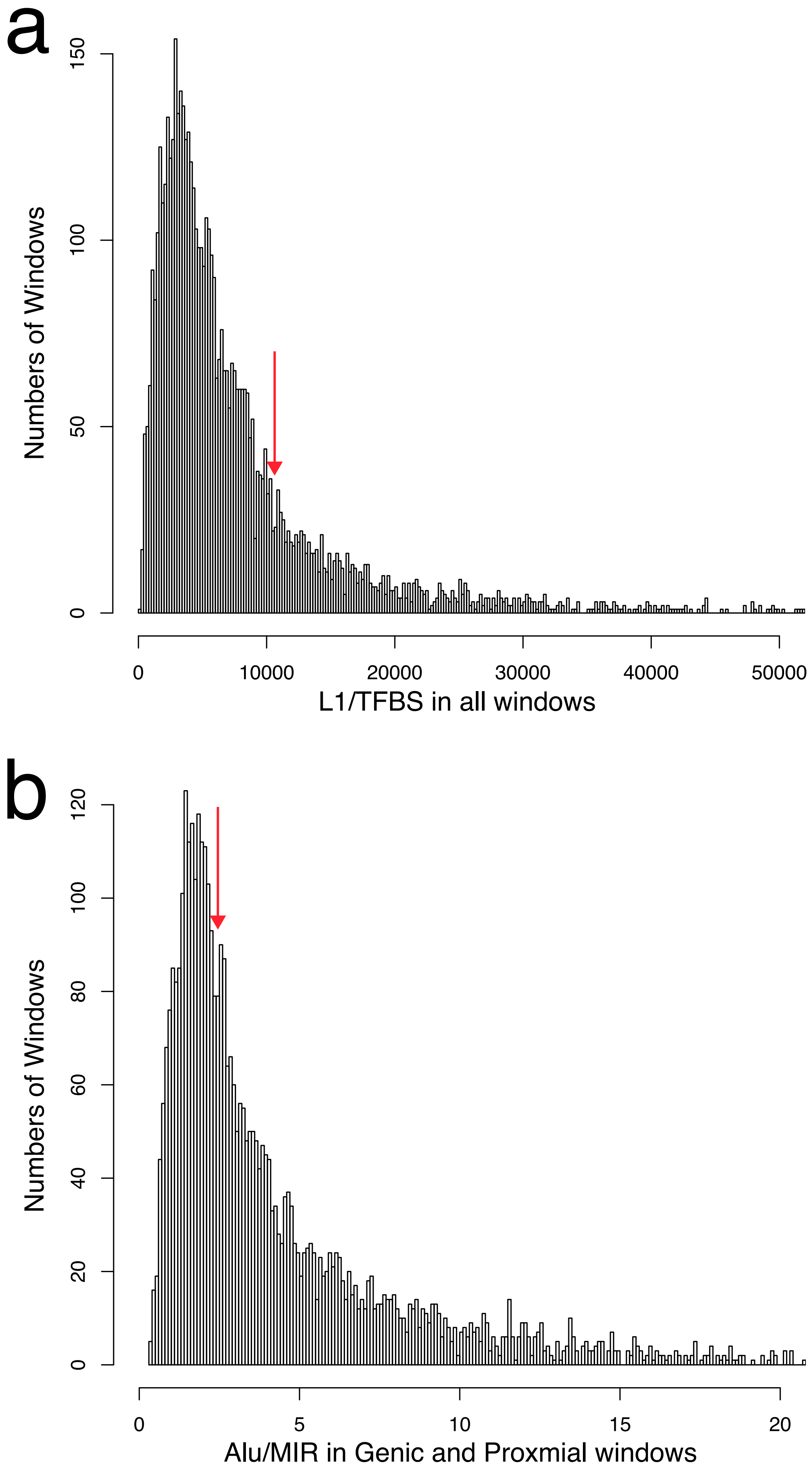

Supplementary Figure S4

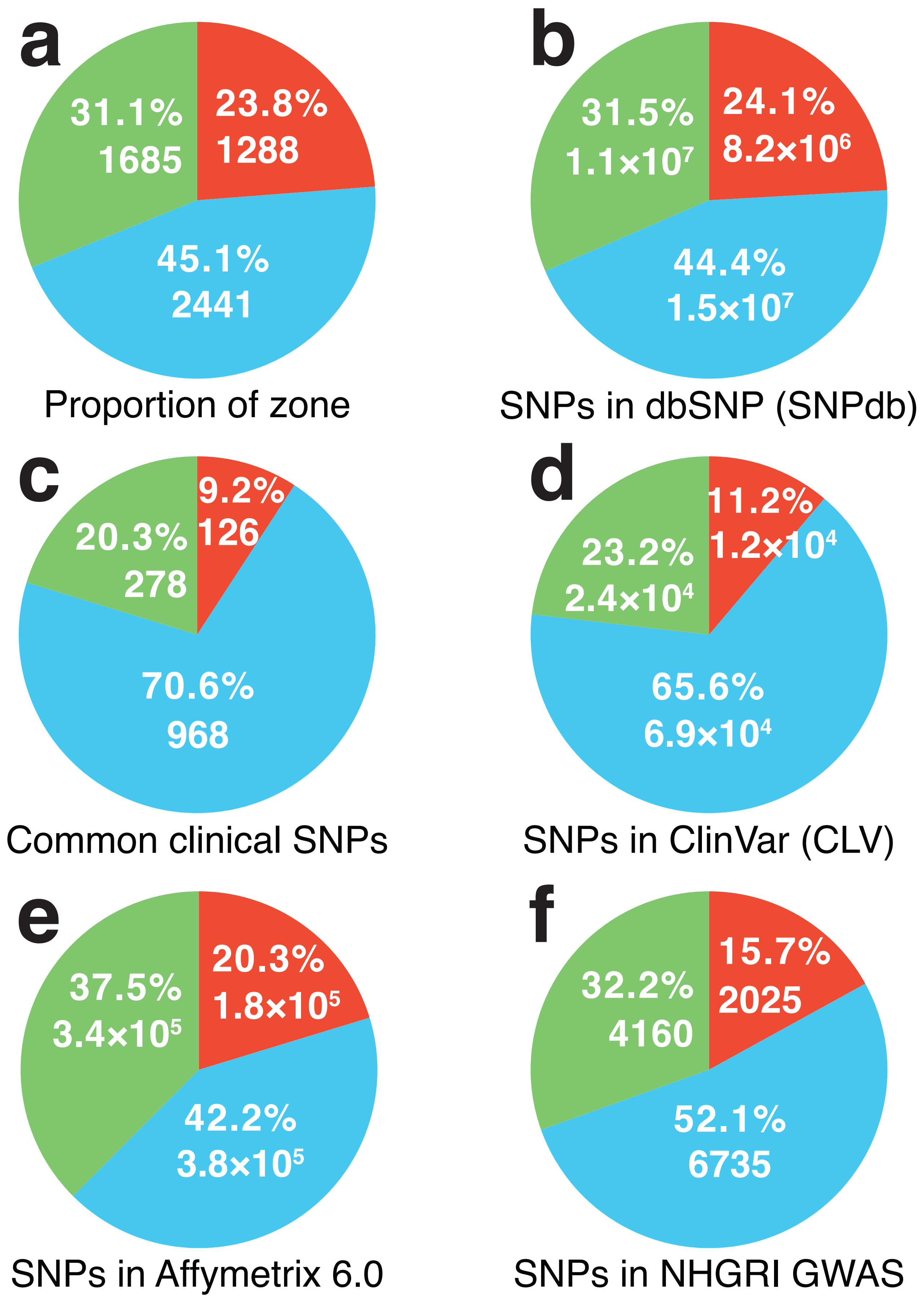

Supplementary Figure S5

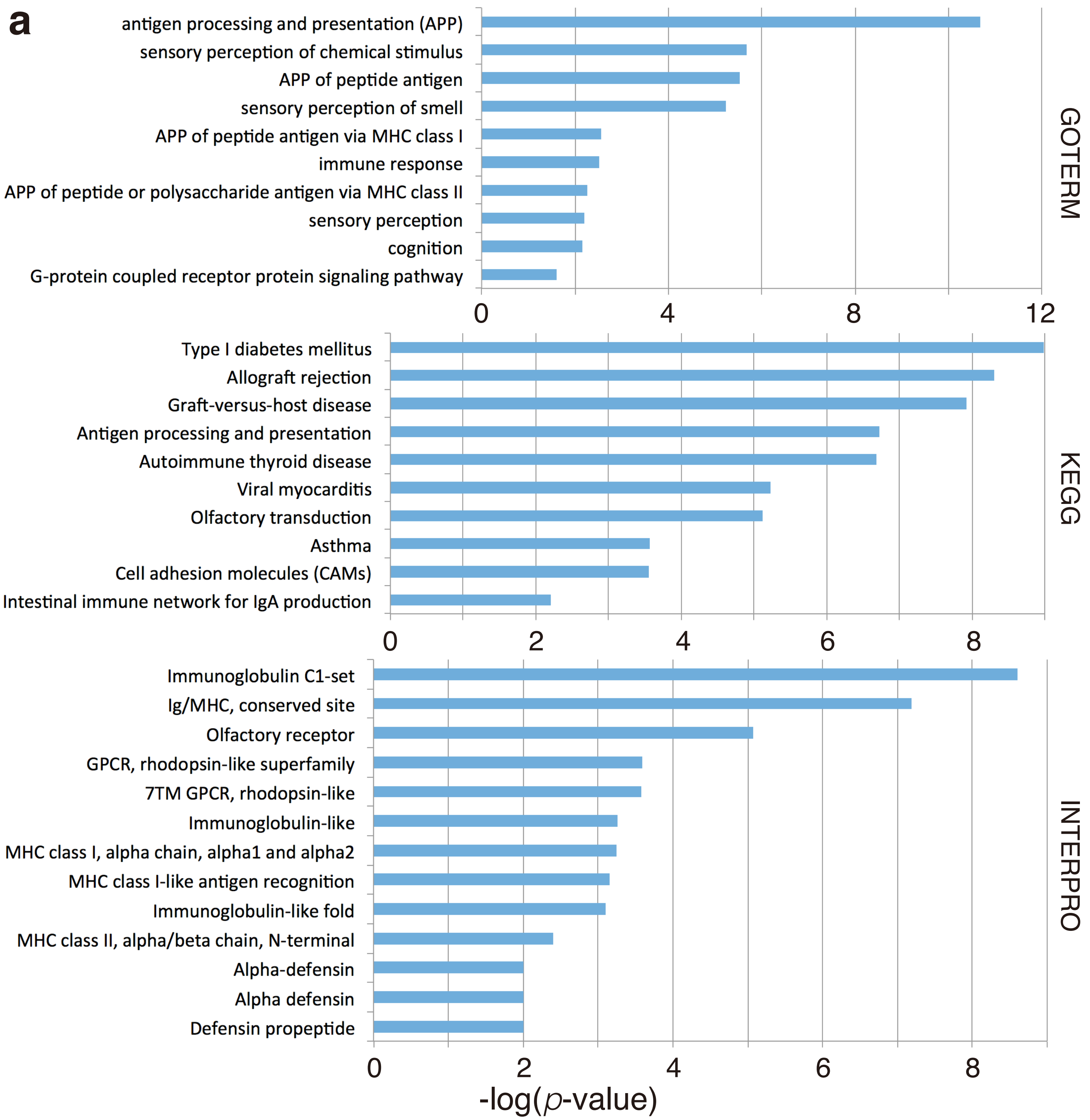

Supplementary Figure S6

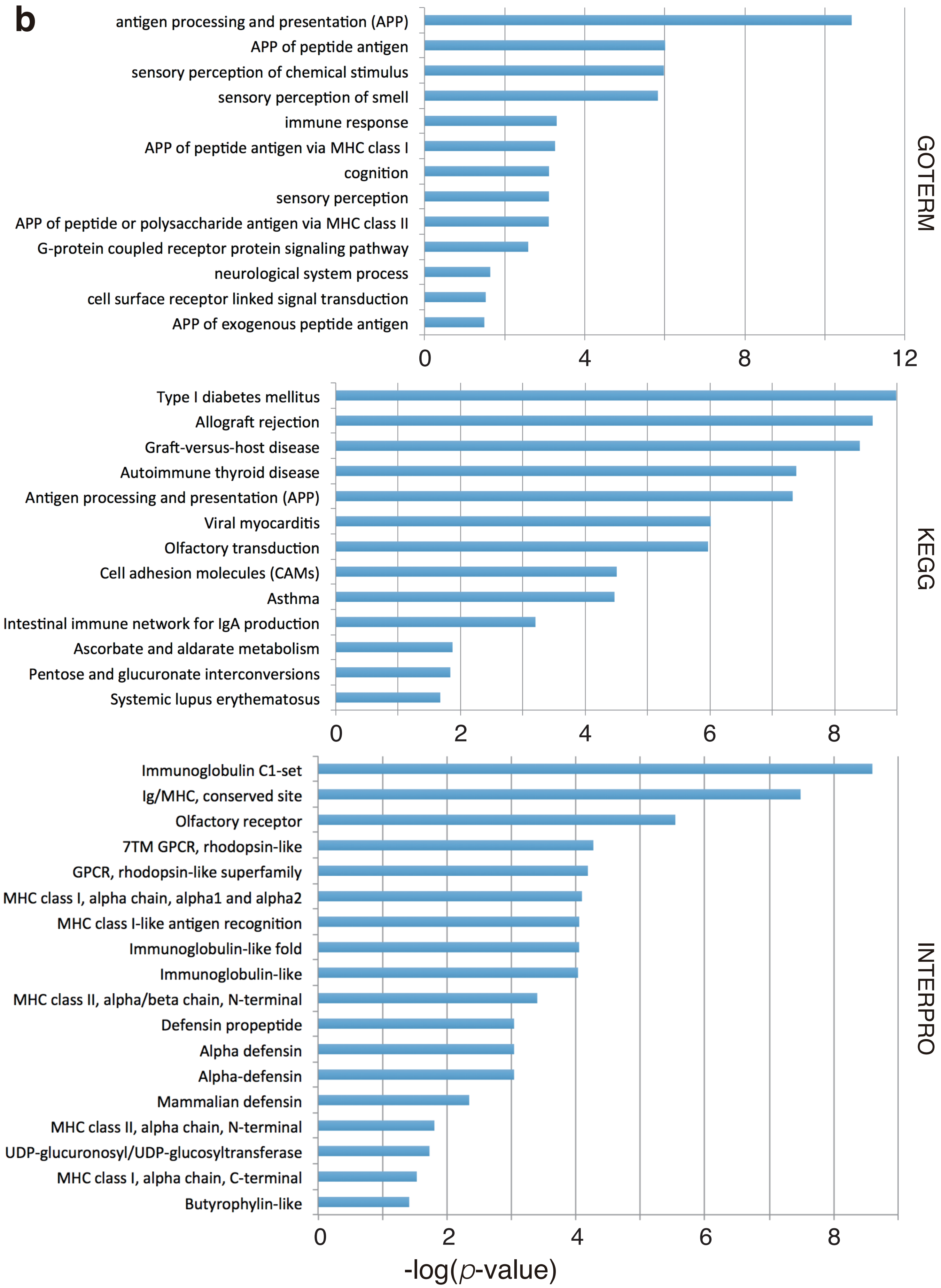

Supplementary Figure S6

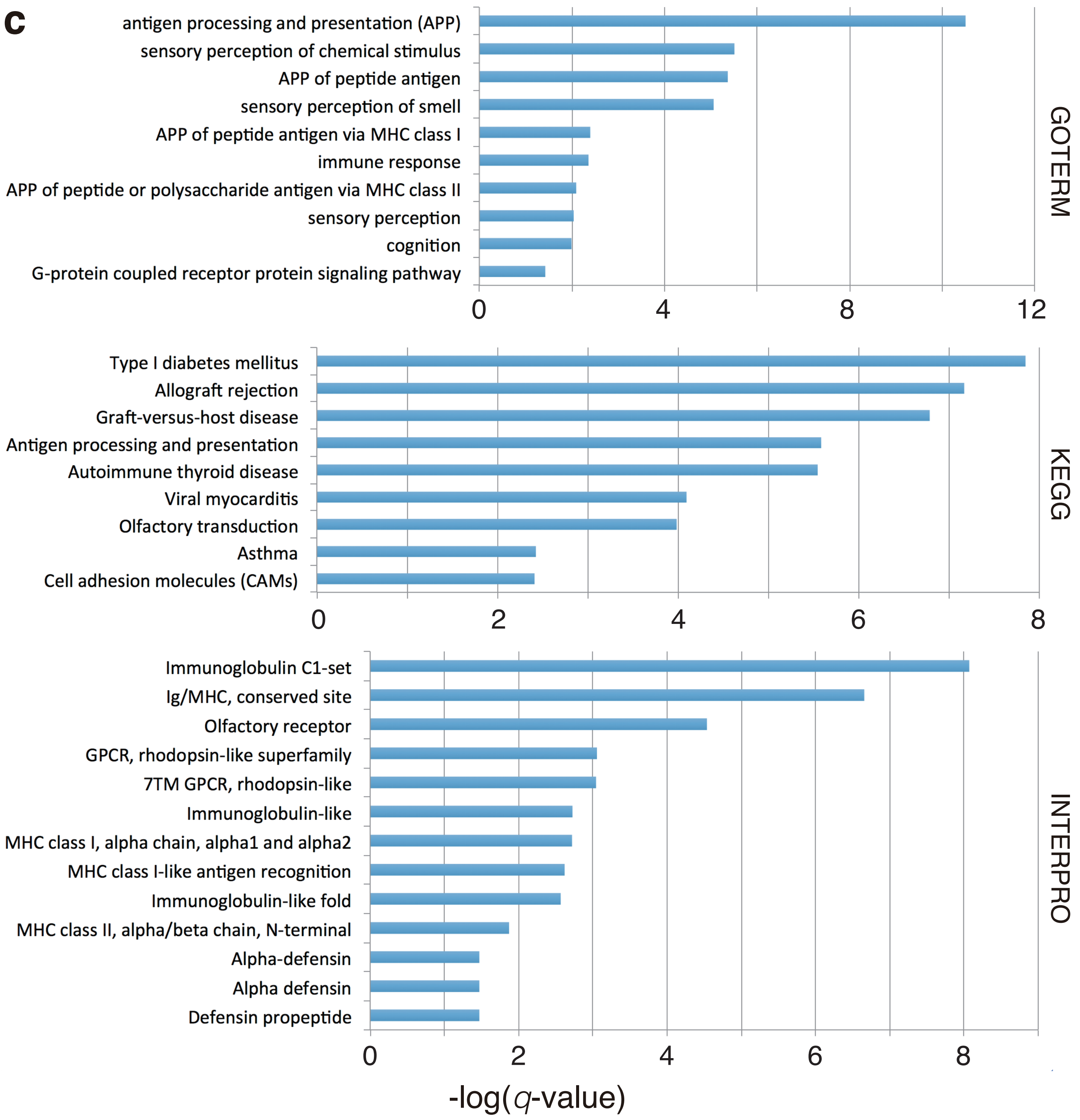

Supplementary Figure S6
